# Weighted Regressions on Time, Discharge, and Season (WRTDS), with an Application to Chesapeake Bay River Inputs^1^

**DOI:** 10.1111/j.1752-1688.2010.00482.x

**Published:** 2010-10

**Authors:** Robert M Hirsch, Douglas L Moyer, Stacey A Archfield

**Affiliations:** * Respectively, Research Hydrologist, U.S. Geological Survey432 National Center, Reston, Virginia 20192; † Hydrologist, U.S. Geological SurveyRichmond, Virginia 23228; ‡ Research Hydrologist, U.S. Geological SurveyNorthborough, Massachusetts 01532

**Keywords:** monitoring, computational methods, statistics, time series analysis, nonpoint-source pollution, nutrients, point-source pollution

## Abstract

A new approach to the analysis of long-term surface water-quality data is proposed and implemented. The goal of this approach is to increase the amount of information that is extracted from the types of rich water-quality datasets that now exist. The method is formulated to allow for maximum flexibility in representations of the long-term trend, seasonal components, and discharge-related components of the behavior of the water-quality variable of interest. It is designed to provide internally consistent estimates of the actual history of concentrations and fluxes as well as histories that eliminate the influence of year-to-year variations in streamflow. The method employs the use of weighted regressions of concentrations on time, discharge, and season. Finally, the method is designed to be useful as a diagnostic tool regarding the kinds of changes that are taking place in the watershed related to point sources, groundwater sources, and surface-water nonpoint sources. The method is applied to datasets for the nine large tributaries of Chesapeake Bay from 1978 to 2008. The results show a wide range of patterns of change in total phosphorus and in dissolved nitrate plus nitrite. These results should prove useful in further examination of the causes of changes, or lack of changes, and may help inform decisions about future actions to reduce nutrient enrichment in the Chesapeake Bay and its watershed.

Hirsch, Robert M., Douglas L. Moyer, and Stacey A. Archfield, 2010. Weighted Regressions on Time, Discharge, and Season (WRTDS), With an Application to Chesapeake Bay River Inputs. *Journal of the American Water Resources Association* (JAWRA) 46(5):857-880. DOI: 10.1111/j.1752-1688.2010.00482.x

## Introduction

Given the importance of water quality to the national and global environment and the efforts being made to improve water quality, there is great value in developing and using data analysis methods aimed at deriving the greatest possible amount of information from the data that are collected, particularly related to changes in water quality over time. Furthermore, it is imperative that the results from these analyses be used to help communicate the water-quality changes that are taking place so that the best information possible is used to guide decisions about future efforts to protect and restore water quality.

The methods of analysis commonly used today were largely developed 20 to 30 years ago (e.g., Lettenmaier, 1976; Hirsch *et al.*, 1982, 1991; van Belle and Hughes, 1984; Gilbert, 1987). Much has changed in the intervening years and this suggests the need to supplement these methods with new approaches to describing and understanding trends in water quality. The changes include: the existence of much larger and longer datasets (e.g., sites with more than 600 observations over a 30-year period), completion of many significant improvements in point-source controls, increased attention to nonpoint sources of pollution, increased interest in the role that groundwater plays in surface-water quality, and public attention to evaluating the progress being made toward resolving ecosystem issues driven by water quality (e.g., Chesapeake Bay or Gulf of Mexico hypoxia). In addition to these changes in the hydrologic and public policy landscape, there have also been changes in the capabilities of computers, and new methods of exploratory data analysis and statistical graphics that can be applied to these issues.

This paper puts forth a new approach for the analysis of long-term surface water-quality datasets. It is designed to be used on datasets with the following characteristics:

1. The number of samples collected at the sampling site is in excess of 200.
2. The period of sample collection is at least 20 years.
3. There exists a complete record of daily discharge values for the site over the entire period being analyzed.
4. All sample analyses are above the laboratory limit of detection (no “less than values”). There is every reason to believe that this constraint can be eliminated, but doing so is beyond the scope of this paper.
5. The samples should be representative of the entire cross-section of the river, such that multiplying the measured concentration times discharge results in an unbiased estimate of flux.
6. At the sampling point, the river should not be so “flashy” that the discharge at the time of sampling is likely to be vastly different from the daily average discharge. This is a matter of judgment, but clearly the method is not appropriate on small streams where discharge is likely to rise and fall by an order of magnitude over the course of a single day. For smaller streams, the method could be extended by using a time step finer than daily.

The method was designed with nutrients in mind but is likely to work well with other major ions and suspended sediment. This paper provides the rationale for the method, illustrates some of the characteristics of water-quality datasets that are discussed in the presentation of the rationale, and defines the mathematical approach and types of products it can produce. In addition, the method described in this paper is used to analyze datasets for total phosphorus and dissolved nitrate plus nitrite for the major river inputs to the Chesapeake Bay. This paper also includes discussion of some of the methodological issues not yet addressed by the methods. It is the authors’ hope that the new approach presented here will lead others toward further advances.

## Seven Desired Attributes of a New Approach for the Analysis of Long-Term Water-Quality Data

Observations about the problems encountered in the scientific study and public policy debates over surface-water quality suggest some desired attributes that a set of new analytical methods should possess. Some of these arise from methodological weaknesses in the standard methods and some arise from unmet needs. Many of the weaknesses mentioned would be very difficult to resolve with smaller datasets.

The total phosphorus record collected at the USGS streamgage 01594440 Patuxent River near Bowie, Maryland, a part of the USGS River Input Monitoring (RIM) program for the Chesapeake Bay, is used to demonstrate the limitations of current methods. These data cover a time period from 1978 through early 2009 and consist of 773 measurements of total phosphorus. The sampling frequency is somewhat variable during the period of record, and in many years is intentionally biased toward sampling at higher discharges, because of their significant role in the transport of pollutants to Chesapeake Bay. At the streamgage site the watershed is 901 km^2^, with a substantial, and growing, suburban population (centered on Columbia, Maryland) but also contains significant amounts of farmland and forests and two large water-supply reservoirs. The historical total phosphorus record reveals a substantial downwards trend in concentration during the past 31 years driven by installation of advanced waste treatment at sewage treatment plants in the basin as well as likely impacts of national limits on phosphate detergents. The profound decrease in concentration over the 31 years makes this dataset an extreme example. Virtually any statistical approach would reveal the trend. This large trend makes the total phosphorus record for the Patuxent River near Bowie, a good case study because it illustrates some of the limitations of traditional trend-analysis methods and shows why enhanced methods are needed to answer some of the questions that policy makers and the public ask about progress in water-quality improvement.

The premise of the work presented in this paper is that there is a need for new methods to analyze long-term water-quality data and these should possess the following attributes:

1. There is a need for methods that focus on description of change. It is a foregone conclusion that in any watershed on the planet, viewed at a time scale of several decades, water-quality conditions are changing. Water quality is nonstationary because of changes in point sources, land use, land-use practices, and atmospheric deposition. Most of the commonly used approaches to water-quality trends are oriented toward hypothesis testing rather than description of an evolving pattern of change. Hypothesis testing certainly has a valid role in the study of water-quality trends. It provides a basis for categorizing individual monitoring sites in a large network, for a specified time period, into three broad categories: those where concentrations are trending up, those where concentrations are trending down, and those where it is “too close to call.” But, for management purposes, there is also a need for tools that will help to elucidate the nature and magnitude of the changes that are taking place. For example, one might want to know: What is the direction and timing of the change? How do today’s conditions compare to those of a decade ago or three decades ago? Has progress toward meeting water-quality goals been speeding up or slowing down or reversing directions? Are improvements happening in some seasons and not others? Are improvements happening at some flow conditions and not others? What can we infer from the answers to these latter questions about the relative roles of point sources, groundwater sources, or storm-runoff sources? The new method presented in this paper provides the means to describe the long-term evolution of water quality at the site, and potentially point to causative mechanisms. Additional work on quantifying the uncertainties of the method should be able to add hypothesis testing to its capabilities. Ideally, one would like to have a unified set of tools that are capable of both hypothesis testing and some of these more descriptive functions.

2. There is a need for approaches that do not assume that the flow *vs.* concentration relation is constant with time. The concept of a flow *vs.* concentration relationship has long played a central role in water-quality data analysis, and rightly so. See, for example, Walling and Webb (1986), Cohn *et al.* (1989), or Chanat *et al.* (2002). In most cases, there is a strong relationship between discharge and the concentration of the constituent of interest. By considering this relationship in the analysis, it becomes possible to substantially reduce the variance in the dataset, thus improving the power of hypothesis tests, the accuracy of estimated trend slopes, or the accuracy of flux estimates. The analytical approach described in this paper builds on the idea that these relationships play a crucial role in explaining significant parts of the variability of water quality, but this approach departs from the older methods in that there is no assumption that the mathematical shape or form of that relationship remains constant over the record. In the more traditional methods (Hirsch *et al.*, 1982; Esterby, 1998; Sprague *et al.*, 2009), water-quality trends are generally defined by changes in the intercept of such a relationship, with no allowance for the relationship to change shape or slope. In this new approach, the relationship is free to change shape and slope in response to the behavior observed in the data.

**FIGURE 1:**
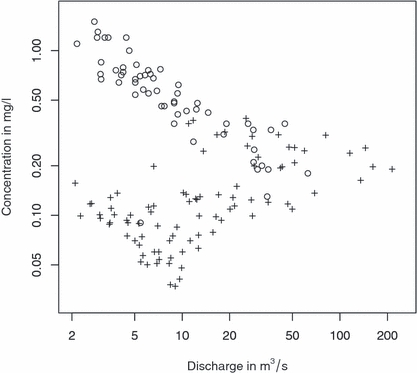
Total Phosphorus Concentration *vs.* Discharge, Patuxent River Near Bowie, Maryland, for Two Time Periods. Data from 1978 to 1984 shown as circles. Data from 2004 to 2009 shown as crosses. Very substantial differences can be observed between the concentration *vs.* discharge relationships for these two periods.

Figure 1 illustrates this point with the Patuxent River total phosphorus data. In Figure 1, the data are arbitrarily divided into an early period (1978-1984, shown as circles) prior to the completion of much of the point-source improvements, and a much later period (2004-2009, shown as crosses) after successful control of much of the point-source loading of phosphorus. The point of this illustration is simply that the shape of the discharge *vs.* concentration relationship has radically changed from the early period to the later period. Because of the dominant role of point sources of phosphorus in the early period, the curve slopes strongly downward, reflecting dilution of the sewage effluent. In the later period, the relationship appears to be quite different. The general slope is upwards, indicating the importance of nonpoint sources of phosphorus, although in the lower range of discharges, below about 10 m^3^/s, there remains an indication of a dilution effect. Any attempt to describe this behavior through the use of a single functional form of a particular slope and shape, even if it allows for a changing intercept over time, will be a poor representation of the data and its lack of fit is likely to introduce spurious trends that are an artifact of the lack of fit. Clearly, any mathematical representation of the behavior of this dataset must accommodate radical changes in the shape of the discharge *vs.* concentration relationship over the span of the dataset.

3. The traditional approaches assume that there is a seasonal pattern in the data that repeats year after year; however, there is also the assumption that this seasonal pattern remains the same throughout the period of record. The timing of peaks and valleys in the pattern may be shifting, due to shifts in dominant processes, and the magnitude of this seasonal cycle also may change over time. There is a need for an approach that makes no assumption that the seasonal pattern repeats in exactly the same cycle over the period of record but rather allows the shape of the seasonal pattern to evolve over time.

**FIGURE 2:**
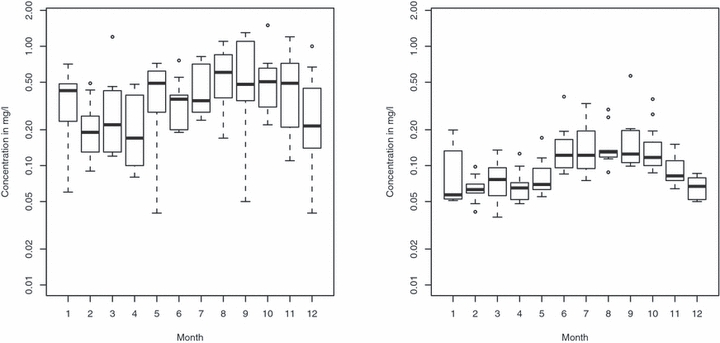
Boxplots of Total Phosphorus Concentration Grouped by Month, for Discharges <15 m^3^/s. The left panel shows the early period (1978-1987) and right panel shows the later period (2000-2008). The amplitude of the seasonal variation is substantially reduced in the later years.

The problem is illustrated by the boxplots shown in Figure 2. Here again the data are divided into two groups (an early period when concentrations were generally very high and a later period in which they were much lower). In this illustration, the data are limited to concentrations from days with discharge <15 m^3^/s (to limit the influence of discharge on the concentration record). The left panel shows the period 1978-1987 and the right panel shows the period 2000-2008. Both panels reveal substantial seasonal variation with the highest concentrations in the summer and early fall months and the lowest concentrations in the winter and early spring months. The key point to be made here is the change in amplitude of the seasonal pattern. In the early period, the difference between the August median and the February median is 0.385 mg/l or a factor of 3.2. In the later period, the difference between the August median and the February median is 0.067 mg/l or a factor of 2.1. In short, whether one approaches the characterization of seasonality as an additive term or a multiplicative term (as would be the case in a model that describes the behavior in log space), the amplitude of the seasonal pattern is substantially reduced in the later years. Here again, a single characterization of the seasonal pattern is likely to create spurious patterns in the data and needs to be avoided.

4. There is a need for an approach that allows the way the trend is described to be driven by the data and not assumed to follow a specific functional form such as linear or quadratic. Trend patterns should also be allowed to differ for different seasons or flow conditions. One of the problematic consequences of traditional methods that use linear or quadratic functional forms is that the addition of new data at the end of the record is likely to affect estimates of trend slope many years in the past. New methods should be designed in such a way that newly collected data will have only minimal effects on the previous description of the trends. New data should only influence interpretations of the recent past, but not affect the interpretations of conditions many years into the past. In the traditional methods, whether parametric or nonparametric, all the data are used to compute trend slopes throughout the entire period of record.

**FIGURE 3:**
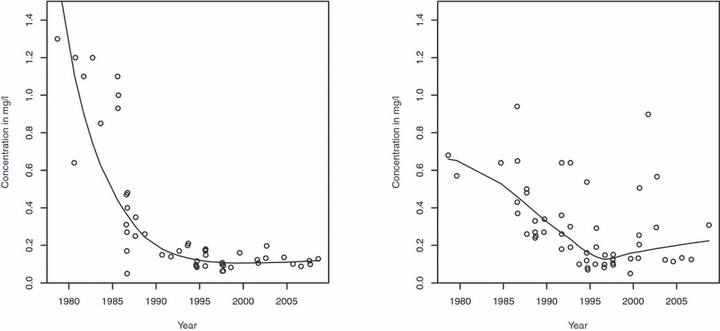
Total Phosophorus Concentrations for August and September *vs.* Time, Patuxent River Near Bowie, Maryland. The left panel shows concentrations in samples for which discharge was <4 m^3^/s, right panel shows concentrations in sample for which discharge was between 4 and 30 m^3^/s. Both panels show local polynomial regression, fitted to the logarithms of the concentration data. The different trend patterns that exist at different ranges of discharge are shown.

Figure 3 shows how the shape of the trend can depart from simple forms. In the left panel, for August and September at low discharges (<4 m^3^/s), we see that from 1978 through about 1995 the trend is steeply downward. It declines by about 1.2 mg/l or 90% over a 17-year period. Since about 1995, the concentrations have stayed relatively constant. Neither a linear nor quadratic function would represent this pattern appropriately. In the right panel, for higher flows (between 4 and 30 m^3^/s), the pattern of change is not as obvious. The decline extends past 1995 and is only a decline of about 0.5 mg/l or about 75%, and then since 1995 there is an indication of a modest increase to the present. The figure makes two points: the first is that trend slopes can change substantially over time, and the second is that the pattern of the trend can be different for different ranges of discharge.

5. The new method should be able to provide internally consistent results describing both concentration and flux. Each of these variables provides important perspectives on the causes and the consequences of water-quality conditions. In most applications, both concentration and flux are important outcome variables. Concentration is the key to ambient quality in the river reach being sampled, whereas flux is the key to conditions in downstream receiving waters (such as reservoirs or estuaries). Of course, concentration and flux are closely related (flux is equal to concentration multiplied by streamflow) but there can be substantial differences in the character of the two kinds of results. For example, a point-source control can dramatically reduce average concentrations because the point-source effluent makes up a substantial part of streamflow on many days, but fluxes may be relatively unaffected by these changes because the bulk of the pollutant moves downstream on high flow days and is determined primarily by nonpoint sources. Trends in concentrations and trends in fluxes (when expressed as percentage changes over time) can be substantially different and there is value in viewing the results both through the metric of concentration and the metric of flux.

6. The method should be designed to provide not only a set of estimates of the time series of concentrations and fluxes, but also a time series of estimates wherein the variation in water quality that can be attributed to the variation in streamflow has been statistically removed. Both kinds of time series (with and without the streamflow variability component) have value and are important for addressing different kinds of questions. The estimated concentration and flux histories can be of great value in understanding the drivers of an ecosystem. They identify times of both high and low concentrations or flux, which may help explain the history of ecological conditions (e.g., algal blooms, fish kills, periods of low light penetration or low dissolved oxygen, changes in aquatic vegetation type or density). Thus, they are useful for testing ideas about the linkage between nutrient inputs and ecological effects in a river, reservoir, lake, or estuary. These records can be very useful as inputs to water-quality and ecological models when they are being tested by hindcasting experiments. However, if the question being pursued is about the effectiveness of control strategies in the watershed, it is very helpful to create records that eliminate the variation in water quality that is driven by streamflow. In this second case, the question is about how the watershed is responding to activities on the landscape such as land-use change, point-source controls, or implementation of best management practices. One way to express the question is to ask, for a given set of hydrologic conditions, are water-quality conditions getting better or worse over time, and how much better or worse? The random variations in streamflow introduce considerable “noise” into the record, making it very difficult to assess progress or lack of progress toward long-term water-quality goals. The goal is to produce records that not only accurately reflect the actual water-quality history, but also eliminate the variation in water quality due to the random variations in streamflow.

7. In addition to providing quantitative estimates of the time histories of concentration and flux, also it would be useful to have within the overall method a set of diagnostic tools that will assist the analyst in understanding the nature of the changes that have taken place over time. It is not enough to say that fluxes have increased by, say 20%, over the last decade. Additional graphical methods should enable the analyst to isolate particular times of year and/or streamflow conditions during which the changes in water-quality conditions are most focused. This kind of analysis should be able to help distinguish among the following potential drivers: changes in point-source effluents, changes in the quality of runoff from the land surface, and changes in the quality of the groundwater that is supporting base flow in the river. This improved understanding of drivers can help to sharpen the focus of strategies for water-quality improvement.

These seven drivers have guided the development of the Weighted Regressions on Time, Discharge, and Season (WRTDS) method described in this report, which is one possible approach to meeting the needs described above. It is intended as a starting point for a new generation of approaches to long-term water-quality data analysis.

## The Method: Weighted Regressions on Time, Discharge, and Season

Weighted Regressions on Time, Discharge, and Season has strong conceptual roots in a class of general models for seasonal time series developed by W.S. Cleveland (Cleveland, 1979; Cleveland and Devlin, 1988; Cleveland *et al.*, 1990) and others over the past 30 years, but it has some distinct differences. The concept is the following. The set of water-quality samples over a period of several decades is likely to have been collected under different sampling strategies. In particular, strategies may have shifted over time from a focus on regularly scheduled sampling (bi-weekly, monthly, or bi-monthly) to a mixed strategy including some scheduled sampling and some event-driven sampling to focus on high flows. It cannot be assumed that a simple statistical analysis of the trend in the sample values will be indicative of a trend in the population of actual concentrations during the period of record. Such an analysis is likely to be strongly influenced by both the changing sampling strategy and the random sequence of high- and low-flow conditions over the period. The alternative strategy used here is to use the sample values to “inform” a flexible statistical model of the behavior of concentrations over the period of record. This flexible model will then be used to make estimates of the concentration for every day of the entire period of record. The model considers concentration to be a product of four components (three deterministic and one random). It simultaneously decomposes the record into these four components:

1. *Trend*: the gradual evolution of conditions from year to year. By definition, the trend component is a smooth function of time, typical of a moving average of a time series where the moving average is over a window of several years duration.
2. *Seasonal*: the annual cycle of variation in water quality that is generally consistent from year to year, although it may gradually evolve over a period of years. By definition, it is a pattern that has a wavelength of a year but does not necessarily follow a set functional form (such as a sine wave). Its amplitude and phase shift and even its shape can change gradually over the years.
3. *Discharge*: the influence that river discharge has on water quality. This relationship is assumed to be relatively smooth. The influence of discharge can evolve over time due to changes in the dominant processes. Downward slopes typify situations where the dilution of point sources or base-flow inputs is dominant; upward slopes typify situations where surface runoff processes dominate. Over the period of record, changes in the relative importance of these processes can result in substantial changes in this relationship, but the changes are assumed to be gradual.
4. *Random*: after removal of the trend, seasonal, and discharge effects, there still remains a substantial amount of unexplained variation in the concentration data. This is the random component. WRTDS does not require a detailed analysis of the random component, although error analysis of WRTDS results requires a set of assumptions about the shape of the distribution of these random errors and about their serial correlation. This paper provides a limited analysis of these random errors, but subsequent work (in progress) will explore this more extensively as a part of a larger effort at WRTDS-uncertainty analysis.

Given these assumptions, we then need a mathematical form for estimating the expected concentrations given any possible combination of (1) time expressed in years (the Trend component), (2) time of year (the Season component), and (3) discharge. The model assumes that these influences are multiplicative. The goal is to estimate daily concentrations (*c*) but, because of the characteristics of the residuals, the model is fit to the natural log of *c*, denoted as ln(*c*), which means that the model can be expressed in additive form.

A regression equation (1) is used to express this relationship, but the method of fitting this regression is the crucial element of the method. 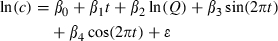
(1)

In Equation (1) the fitted coefficients are the *β* values, *c* is the concentration, *Q* is discharge, *t* is the time in years, and *ε* is the unexplained variation. This method departs from the more common approaches such as those used by Cohn *et al.* (1989) in that the parameters of this equation are estimated for every combination of *Q* and *t* values where estimates are required for the particular result or graphic of interest. The functional form of Equation (1), which is linear in *t*, linear in ln(*Q*), and sinusoidal on an annual period, does not imply that their coefficients apply throughout the entire domain of the data, but rather that they are useful approximations for describing relationships over a limited portion of the domain. A weighted regression estimation system that can yield an expected value of *c* for any given combination of *Q* and *t* is at the center of the WRTDS method. Define *Q*_o_ as the discharge (in cubic feet per second) and *t*_o_ as the time (in years) for which we want an estimate of *c*. The logic of the estimation method is that we will estimate the parameters of Equation (1) using weighted regression where the weights on each observation are based on the relevance of that observation to the estimation point (*Q*_o_, *t*_o_). The relevance of each observation is defined here by a distance between the observation (*Q*_*i*_, *t*_*i*_) and the estimation point. This distance has three dimensions. The first is distance as measured by the difference between *t*_o_ and *t*_*i*_ known as the “time distance.” The second is distance as measured by the difference between the time of year at *t*_o_ and the time of year at *t*_*i*_ known as the “seasonal distance.” The third is distance as measured by the difference between ln(*Q*_o_) and ln(*Q*_*i*_) known as the “discharge distance.”Cohn (2005, p. 9) also used a windowed approach to the regression for some of the same reasons that motivate the WRTDS method. Cohn’s approach differs from WRTDS in that it did not use weighted regression and is applied in one dimension rather than the three dimensions used in WRTDS.

**FIGURE 4:**
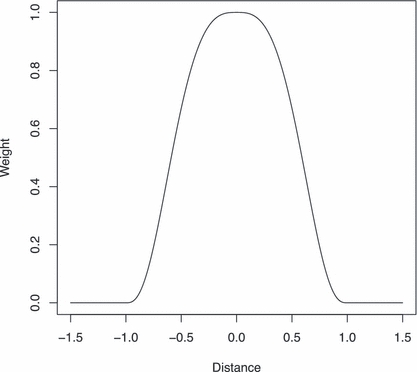
Tukey’s Tricube Weight Function (Tukey, 1977), With the Half-Window Width (*h*) Set to 1.

For all three of the distance measures, we will use the same general weighting function. It is the “tricube weight function” originally defined by Tukey (1977). The form of the tricube weight function is: 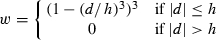
(2)where *w* is the weight, *d* is the distance from the estimation point to the data point, and *h* is the half-window width. The function looks somewhat like a normal distribution, but is a bit flatter at the top and rather than approaching zero asymptotically, this function goes to zero beyond the edges of the window. It is shown in Figure 4, for *h* = 1.

Note that at a distance of half the half-window width, the weight is 67% as large as it is in the center of the function, and at a distance of three quarters of the half-window width the weight is only 19% as large as it is in the center of the function. This approach results in more stable and robust results than a simple moving window approach, where weights are 1 where distances are small and go to zero abruptly where distances are large. As the simple window moves through the sample space, the model coefficients can change abruptly as particular highly influential observations enter or leave the window potentially resulting in abrupt changes in model estimates over short spans of time. With the smoothing approach to setting weights used in WRTDS, the influence of any given observation declines gradually to zero as distances become greater. Thus, the response to influential observations changes gradually across the domain and the overall predictive model never changes abruptly from one point in the domain to another nearby point.

The overall weight for each data point to be used in the weighted regression is determined as the product of the three component weights. Note that when any one of the three weights goes to zero, the overall weight is zero. The weights are implemented as follows:

1. *Trend*: For this weight, distance is measured in terms of time in years. The half-window width is specified in years. Experience thus far has suggested that 10 years is a suitable half-window width. With this width, those observations that are more than about six years from the center of the window have weights that are less than half of the weights at the center of the window. Experimentation has demonstrated that narrower time windows result in high-frequency year-to-year variations in estimated concentrations (holding discharge and season fixed). The assumption used in WRTDS is that the trend component changes in a smooth pattern over time, rather than rising and falling over periods of a year or two. This is explored below in the section on method sensitivity.
2. *Seasonal*: The logic of the seasonal weights is that those data points that are close to the time of the year for which we want an estimate should have high weights, but those that are in a different part of the year should have low weights. For example, January data are of little relevance in predicting July values. This is implemented as follows. If *t*_o_ is the time variable for the estimation point expressed in units of years, then, for example July 1, 2009, would be 2009.5. The time variable for the sample point expressed in units of years is *t*_*i*_. Then *d*_s_ (the “seasonal distance”) is computed as follows 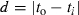
(3)
3. Define *r*_u_ as *d* rounded up to the next integer and *r*_d_ as d rounded down to the next integer. The seasonal distance, *d*_s_ is the difference in time of year between *t*_o_ and *t*_*i*_, which can be expressed as 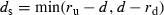
(4)
4. The seasonal weight can then be computed using *d*_s_ as the distance term. For example, if the estimation point is July 1, 2009, and the sample point is July 1, 2000, the value of *d*_s_ = 0. This means that, from a seasonal perspective, this sample value is highly relevant for estimation. For the same estimation point, if the sample point was November 15, 2000, the value of *d*_s_ = 0.38. This means that, from a seasonal perspective, this sample has very limited relevance. Experience has indicated that a half-window width of 0.5 works well. In this case, the only dates with zero weight are those exactly a half a year from the estimation point, but all dates more than about 3.6 months from the estimation point have weights <0.5. In the two cases above, the July 1 sample would have a seasonal weight of 1 and the November 15 sample would have a seasonal weight of only 0.19.
5. *Discharge*: This distance is measured in terms of ln(*Q*), where *Q* is the daily discharge. Experience to date has suggested a half-window width of 2.0 for smaller rivers such as the Patuxent River near Bowie. That means that we only use data where the flow is within two natural log cycles of the discharge at the center of the window. For example, if the window was centered on 100 m^3^/s (*Q*_o_ = 100), then the window would have nonzero weights for days with flow values between 13.5 and 739 m^3^/s. The days with weights of >0.5 would lie between about 30 and 332 m^3^/s. We might expect that on much larger rivers, where the range of the logarithm of daily discharges tends to be smaller, that the appropriate half-window should be smaller.

**FIGURE 5:**
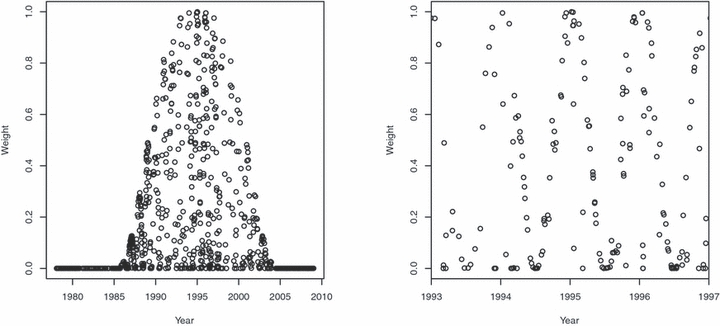
Weights Computed for the Patuxent River Near Bowie, Maryland, for January 1, 1995, and a Discharge of 10.5 m^3^/s (the long-term median). Note the general bell-shaped form of the weights. The right panel is an expansion of the four years centered around January 1, 1995. It demonstrates the seasonal patterns of the weights, with a maximum around January 1 of each year and a minimum around July 1 of each year. Note a few particularly low weights in January of 1996. These weights are very low because these samples were collected during a flood, resulting in a very low discharge weight, and thus a very low overall weight.

The overall weight for each data point is the product of these three individual weights. Thus, a data point being a “long distance” from the estimation point in any one of the three dimensions (time, season, or discharge) will disqualify it from being a part of the regression, or at least greatly diminish its importance. The half-window widths chosen may seem overly liberal, but it is this multiplicative screening process that makes that necessary. The use of wide half-window widths is intended to prevent an overly narrow description of the relevant “neighborhood” for estimation, which could result in rapid variation of model coefficients across the range of *Q* and *t* values. The net effect of this approach is shown in Figure 5, which plots the overall weight for every one of the 773 observations in the Patuxent River near Bowie, total phosphorus dataset, when the estimation point is set at January 1, 1995, and the discharge is set at the long-term median of 10.5 m^3^/s. Note that the figure has an overall “bell shape” based on the time window. We can also see a kind of vertical stripe effect with higher weights near January 1 of each year and lower weights around July 1 of each year.

The fitting procedure has a safety check to assure that the estimates are based on a sufficient sample size. At some estimation points, the windows may be so restrictive that very few data points end up having nonzero weights in the estimation of this four-variable weighted regression equation. This is most likely to happen at the “edges” of the estimation space near the beginning or end of the record and at the most extreme ends of the flow distribution. Given that a large number of weighted regressions are used in any application of the method, it is important that the size of the sample be relatively large to guard against regressions that predict highly extreme concentrations because the sample used in one particular regression contained a few unusual and highly influential observations. Common rules of thumb for multiple regression analysis suggest that for a four variable model such as that being used here, a total of 82 observations might be considered adequate (see, e.g., Tabachnick and Fidell, 1996). Given that these are weighted regressions and some of the samples may be used but only carry a very low weight, an arbitrary determination was made to require at least 100 observations with nonzero weights. This is implemented in the software as follows: if there are 100 or more observations with nonzero weights, then the weighted regression is run; however, if there are fewer than 100 observations, then all three of the half-window widths are increased by 10% and new weights are assigned to each value. If there are at least 100 observations with nonzero weights, then the weighted regression is run. Otherwise, the software iterates through this widening process until they are wide enough to have at least 100 observations with nonzero weight. Even with this provision for a minimum sample size for each regression, there are instances where at the edges of the sample space there may be a bit of “sawtooth” behavior seen in plots of estimated concentrations *vs.* time or discharge. This behavior is a result of the window-widening process. It can affect the appearance of some of the graphics that are presented in the next section, and often it serves as a reminder to the analyst to restrain the estimation process to only the parts of the time, discharge, and season space that are realistic. The setting of this minimum sample size, as well as the selection of window widths, is an appropriate subject for further exploration. Experiments run with substantially narrower window widths than those suggested here can lead to changes of 10% or even slightly more in terms of summary statistics for individual years or trend slopes over periods of five or fewer years. At time scales of 10 years or more, however, these choices of windows appear to have very little influence on the size of the observed trends.

In summary, this weighting process results in a set of weights on every observation in the dataset, based on the selected values of *t*_o_ and *Q*_o_. The weighted regression is run and then the fitted coefficients are used to estimate the expected value of ln(*c*) for that specific time and discharge. However, the results desired are not the expected value of ln(*c*) but rather, the expected value of *c*. The problem of re-transformation bias is well understood and several methods are available to remove this bias (Bradu and Mundlak, 1970; Cohn *et al.*, 1989, 1992). The simplest and possibly the most robust form of transformation bias correction is the smearing estimator, developed by Duan (1983). The bias correction for WRTDS is implemented as a weighted form of the smearing estimator. If *Y* is the expected value of ln(*c*) for any given discharge and time, then the unbiased estimate of *c* for that discharge and time is 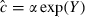
(5)where 
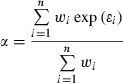
(6)and *ε*_*i*_ is the *i*th residual from the weighted model, *n* is the total number of observations in the dataset, and *w*_*i*_ is the weight on the *i*th observation.

The WRTDS approach provides a nearly unbiased and relatively free-form approach to estimating the expected value of concentration for any given date and discharge. A wide range of graphical approaches are possible (and several of them have been implemented) to explore the WRTDS model’s representation of the evolving behavior of the system. The following section uses one of these graphical approaches in an overview of results and model sensitivities.

## Model Results and Sensitivities

**FIGURE 6:**
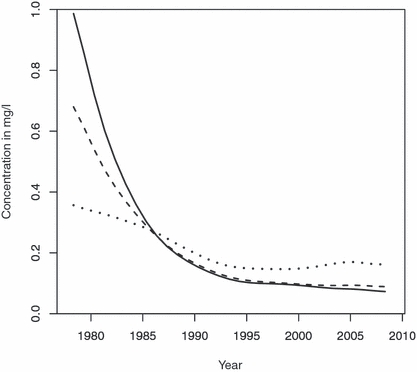
Estimated Concentration of Total Phosphorus, Patuxent River Near Bowie, Maryland, Evaluated at May 1 of Each Year for Discharge Values of 5 (solid), 9 (dashed), and 30 (dotted) m^3^/s. Note the crossover of the curves around 1986. Prior to that time, concentration tended to fall with increasing discharge. After that time, concentration rose with increasing discharge.

One approach to exploring the nature of the changes in water quality in the record is to select a specific discharge and a specific time of year and use WRTDS to compute the expected concentrations on that date for every year in the period of record for that specified discharge. These expected concentration values can be graphed as a function of time. Three different discharge values can be plotted on the same graph. Figure 6 is an example of this. The selected date is May 1 and three specific discharges are selected and plotted on this figure. They are 5 m^3^/s (approximately the 10th percentile flow for May 1), 9 m^3^/s (approximately the 50th percentile flow for May 1), and 30 m^3^/s (approximately the 95th percentile flow for May 1). Figure 6 shows that the most substantial decreases in concentration have taken place at the lowest discharge, and that those decreases seem to be continuing to the present, although the decline was the steepest in the 1980s. At the median discharge, the decreases are still substantial, although they started from a lower initial value they have ended up being quite close to the values for the lowest flows. In contrast to these results, at a high discharge, the decrease has been much less substantial and actually shows some indication of increases in the last 10 years of the record. The reason for these differences is that the point-source controls, that were so significant in terms of low flow conditions, have less effect at the higher discharges. For example, the results shown in Figure 6 indicate that between May 1, 1980, and May 1, 2007, for a discharge of 5 m^3^/s, the expected concentration decreased from 0.71 to 0.076 mg/l, a decrease of 0.63 mg/l or 89%. But, for a discharge of 30 m^3^/s, the expected concentrations for those dates decreased from 0.33 to 0.16 mg/l, a decrease of 0.17 mg/l or 54%. Thus, expressed either in absolute terms or percentage terms, the decrease over that 27-year time span was much larger for a low discharge than for a high discharge.

**FIGURE 7:**
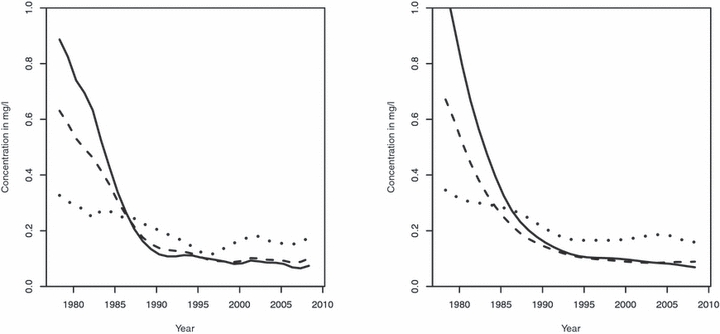
This Figure Presents the Same Analysis as Shown in Figure 6, Where the Half-Window Widths Were 10 Years, 2 Log Units of Discharge and 0.5 Years for the Seasonal Window. The three discharge values are 5 (solid), 9 (dashed), and 30 (dotted) m^3^/s. In the left panel, the half-window width for time has been reduced to five years. In the right panel, the half-window width for discharge has been reduced to 1 log unit.

Exploring the question of the sensitivity of the results to the window widths, Figure 7 shows two examples of variations on the results shown in Figure 6. Figure 7 shows the rapid, year-to-year fluctuations that arise when the time half-window width is reduced from 10 to 5 years. The implicit assumption of the WRTDS approach is that there is an underlying “behavior” of the system that evolves rather slowly over time and represents the net effect of many small changes in many small portions of the watershed. If the curves such as those shown here are fluctuating at time scales of a year or two, the assumption is that the method is “overfitting” the model and is actually tracking the random outcomes of the set of samples in the dataset rather than following a pattern that is representative of underlying changes in watershed. The right panel in Figure 7 shows the results of a change in the discharge window from 2 (as shown in Figure 6) to 1. Here, the differences are less clear and there is no particularly compelling argument that can be used to choose one half-window width in preference to the other. This topic of “optimal” window widths is an important topic for further study. Such an evaluation requires the use of datasets that are substantially denser than the ones used in this study so that error properties associated with different window widths can be evaluated. What can be said at this point from a significant amount of testing is that, over a wide range of possible window widths, the general nature of the results is quite consistent. The estimates of average concentrations and direction and slope of the trends are consistent. The differences are in the extent of smaller variations at time scales of about five years or less. These three examples (Figures 6 and 7) demonstrate results that all tell similar stories about the three decades of change that has taken place in the watershed.

One way to consider the usefulness of the WRTDS approach is to explore the residuals from use of the model in comparison with other reasonable approaches. The WRTDS method was used to compute estimates for the 773 days on which actual sample concentration values are available for total phosphorus. These residuals were computed in log space. The residual is the difference between the actual ln(*c*) and the WRTDS estimate of ln(*c*) (the bias correction was not used in these calculations). These residuals follow a distribution that is quite symmetrical, although the distribution has tails that are heavier than those of a normal distribution. An equivalent *R*^2^ value for these estimates is 56%. That is, the WRTDS explains 56% of the total variance in the dataset. If we compare that with a model based on Equation (1), with the coefficients all fixed throughout the record, the *R*^2^ is 35%. Thus, the technique results in a substantial reduction in variance, although it must be recognized that there remains a very large amount of unexplained variance in these data (and the other datasets explored later in this study). Appendix S1 reviews the error properties of WRTDS compared with five simpler models for this dataset and for dissolved nitrate plus nitrite concentrations in samples collected at the USGS streamgage 01491000 Choptank River near Greensboro, Maryland (discussed later in this paper). In both cases, the WRTDS equivalent *R*^2^ value is substantially higher than simpler models and the WRTDS better reflects the nature of the trend that is taking place.

### Computation of Concentration and Flux Histories

The ultimate product of the WRTDS is a time series of estimated concentration and flux for the entire period of record. The first approach to this is to compute these histories using the actual history of discharge that happened over the period of record. In this approach, WRTDS is used to estimate an expected concentration for every single day of the record by using the actual daily discharge for that day and the time variable representing that day. To save on computational effort, rather than doing a new estimate of the weighted regression equation for each day, a matrix of regression results is created and the estimate for any given day is determined using linear interpolation of the results stored in this matrix. This matrix of regression results is three dimensional. The first dimension is time in years. The second dimension is time in months from 1 to 12. The third dimension consists of 12 levels of discharge equally spaced in log space spanning the full range of observed daily discharge values in the record. This process of interpolation in this three dimensional matrix of results is used to estimate concentration values for each day of the study period. The computational savings from using this interpolation method is rather small for the purposes described here, but in a subsequent step in the analysis (flow-normalization, discussed below), it reduces the number of weighted regression estimates required by about a factor of 80 for a record of 31 years. Analysis of the interpolation method compared to running the weighted regression analysis for each day reveals that the interpolation adds a small amount of error. When aggregated to annual values, there is almost always less than a 1% difference between the interpolated results and the more direct approach.

**FIGURE 8:**
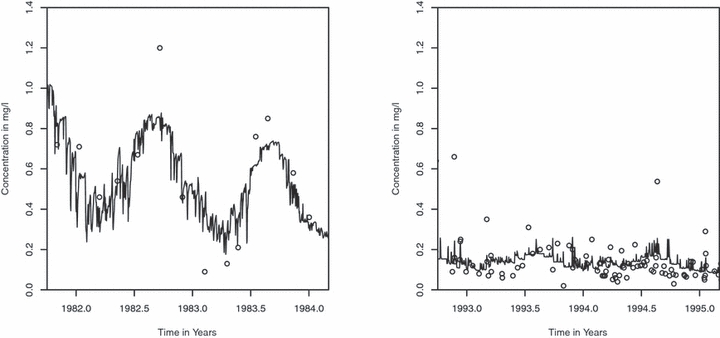
Observed Concentrations (circles) and Predicted Concentrations (lines), Using the WRTDS Model, for Total Phosphorus Concentration, Patuxent River Near Bowie, Maryland, for Two Different Time Periods: November 1981 to January 1984 (left panel) and November 1992 to January 1995 (right panel).

Figure 8 provides two examples of this estimation process. Each graph covers a period of slightly more than two years and shows the observed concentrations (as circles) and the daily predicted values using WRTDS. The left panel shows a period when the concentrations were high but were coming down as a result of the sewage treatment plant upgrades. The right panel (drawn to the same scale) shows the pattern about a decade later. One thing that is immediately clear from these two panels is that WRTDS generally matches the substantial decline in concentration. They also show how pronounced the seasonal pattern was in the early period and how subdued it became in the later period. In addition, the panels show that the predicted values are always less variable than the observed concentrations. During periods when the model predicts low values, the actual values are likely to be even lower and in periods when the predictions are high the actual values are often higher. This is to be expected for any regression-based estimation scheme. These graphs are reminders that the estimates always “regress to the mean” but should be unbiased estimates overall. Thus, a set of WRTDS estimates should not be used to estimate the frequency of exceedance of a threshold, although it can be used in the process of making such estimates.

These concentration estimates computed as described above can then be used to make flux estimates: 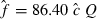
(7)

Here, 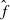 is the expected value of flux, in kg/day, 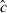 is the expected value of concentration in mg/l, *Q* is daily discharge in m^3^/s, and 86.40 is the unit conversion factor. These time series of estimates can then be summarized into time series of monthly averages and then into annual averages either of which can be graphed *vs.* time.

**FIGURE 9:**
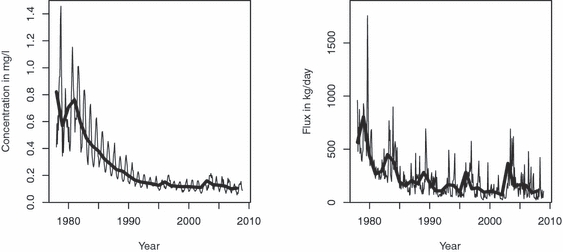
Left Panel Shows the Estimated Monthly (light line) and Annual (heavy line) Estimated Concentrations for Total Phosphorus for the Patuxent River Near Bowie, Maryland. The right panel shows the monthly and annual estimated flux.

Figure 9 shows the estimated monthly and annual concentration (left panel) and flux (right panel) for the Patuxent River dataset. Several features are noticeable in these panels. They both show substantial declines over time. Both show a strong seasonal pattern, but in the case of concentration, the seasonality and overall trend in the annual averages is much more regular whereas the flux records show much more random variation from the overall decline. Both records show the effect of the very high discharge years of 1996 and particularly 2003, although these effects are much more pronounced in the flux record than in the concentration record.

### Flow-Normalization

As mentioned in Item 6 of “Seven desired attributes of a new approach for the analysis of long-term water-quality data,” the method should produce not only estimates of concentrations and fluxes for the entire period of record, but also histories of concentrations and fluxes in a manner that removes the random variations in these quantities that arise from the random variations in discharge that took place over the period of record. For example, consider a hypothetical watershed in which there have been no changes in land use, land-use practices, point-source loadings or any other human-driven factors during the period of record. In this example, consider the possibility that the last few years of the record were drought years, and the constituent of interest was one that had lower concentrations at lower discharges, then it is likely that the last few years of the record would be ones of low concentration and very low flux. We would want our method of analysis to recognize that the observed changes in concentration or flux, although very real, are not indicative of any actual improvement in watershed conditions, and that when the drought ends concentrations and fluxes will return to higher levels. Thus, when our interest is in the progress being made in the watershed toward the attainment of water-quality goals, we need a method that will assure us that the trend we perceive is a result of a change in the way the watershed responds to the full range of hydrologic conditions and not simply a result of the temporal pattern of hydrologic conditions that happened to have taken place in our period of record. Accomplishing this motivates the development of the “flow-normalization” procedure described below.

Flow-normalization eliminates the influence of the temporal pattern of discharge, by viewing the discharge on any given day as a random sample of the discharges that might have taken place on that day. Thus, the method requires some means of estimating the probability distribution of discharge values for that day. Flow-normalization uses the actual historical sample of discharge values for a given day, with each historical value being assigned an equal probability of happening in any given year. This empirical approach avoids the challenges of developing a stochastic streamflow model to generate a set of potential streamflow realizations. The discharge that occurred on any given day of the record is assumed to be one sample from the probability distribution of discharge for that particular day of the year.

Using the Patuxent River example, for any given date, such as April 20, 2003, the flow-normalized concentration estimate assumes that all 31 of the April 20 discharge values in the record (from 1978 to 2008) were equally likely to have happened on April 20, 2003. Thus, to compute the flow-normalized estimate of concentration for April 20, 2003, the method estimates 31 values of concentration using the WRTDS model with the time variable set to April 20, 2003, but with the discharge variable set to each one of the 31 historical discharge values for April 20. The “flow-normalized concentration” is simply the mean of these 31 estimated concentration values. Similarly, the “flow-normalized flux” is the mean of 31 flux values computed by using the WRTDS model.

Implementation of the approach, although being conceptually simple, is computationally intensive. For example, for the 31-year record on the Patuxent River, there are 11,323 days for which flow-normalized concentration estimates are made, but because each of these is an average of 31 values, the WRTDS model is producing about 351,000 (∼31 × 11,323) individual estimates of concentration. It is because of this highly repetitive process that the method has been implemented as an interpolation scheme using a set of 4,464 weighted regression estimates (for this particular record length) rather than calculating the full set of about 351,000 weighted regression estimates.

**FIGURE 10:**
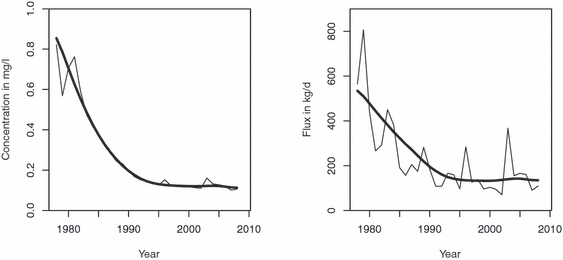
The Left Panel Is the Annual Average Estimated Total Phosphorus Concentration (light line) and the Flow-Normalized Annual Average Estimate (heavy line), for the Patuxent River Near Bowie, Maryland. The right panel contains flux estimates. In both cases, the annual estimates (light line) are the same as those plotted as the heavy line in the two panels of Figure 9.

The flow-normalized concentration and flux estimates can be summarized into time series of monthly averages, which, in turn, can be summarized into annual averages. The annual averages of flow-normalized estimates can be superimposed on a graph of the annual estimates (Figure 10). The resulting flow-normalized annual concentration and flux histories are very smooth temporally because they eliminate all the variation that is due to the random variation in streamflow. These results should provide a much clearer indication of true progress (or deterioration) toward (or away from) the achievement of water-quality goals. What is meant by “true progress (or deterioration)” is change in water-quality drivers such as land use, land-use practices, or point-source loading. Because the flow-normalized records are not driven by random variations in streamflow and because they are much more stable than the actual record of water quality, they are appropriate to use when computing changes over time.

For our example case, one can make statements such as the following. For flow-normalized concentration, the change over the period 1980 through 2008 has an average slope of −0.026 mg/l per year, or −3.1% per year. For flow-normalized flux, the average slope is −14 kg/day per year, or at an average rate of −2.7% per year. Considering the substantial change in the rates of decrease evident in Figure 10 and concerns that decision makers may have about recent rates of improvement, one might want to express these rates of change in terms of individual time intervals. Table 1 is an example of such a display, using periods of about a decade in length. It conveys the dramatic decline in the rate of change of both flow-normalized concentration and flow-normalized flux over time. In the case of the flux results, there is a slight indication of a trend-reversal in the 2000 through 2008 period. The indications of this reversal are rather weak, but they certainly suggest that fluxes are likely not continuing to decline at rates such as those that were observed in previous decades.

**TABLE 1:** Trend Slopes for Flow-Normalized Concentration and Flux, for Various Time Periods, Total Phosphorus, Patuxent River Near Bowie, Maryland.

	Concentration Results		Flux Results	
Time Span	Slope (mg/l per year)	Slope (% per year)	Slope (kg/day per year)	Slope (% per year)
1980-1990	−0.051	−7.2	−28	−5.9
1990-2000	−0.0076	−3.9	−6	−3.2
2000-2008	−0.00094	−0.8	+0	+0.2

In general, annual average concentrations (actual or flow-normalized) will tend to reflect conditions over the many days of low to moderate flow, and these are strongly determined by point sources and base-flow contributions. Conversely, annual average flux values (actual or flow-normalized) will tend to reflect conditions on the relatively few days of the year with very high streamflow, and thus they are strongly determined by nonpoint-source runoff-related contributions. Because the flow-normalized values remove a substantial amount of variability from the annual averages, they are suitable for evaluating long-term trends. In general, a trend in the annual average flow-normalized concentration is more indicative of trends in point-source or base-flow contributions, and a trend in the annual average flow-normalized flux is more indicative of trends in nonpoint-source runoff-related contributions. Thus, the results seen in Table 1 suggest continuing modest reductions in point-source loadings but modest increases in loadings at higher discharges (associated with nonpoint sources). Tables and graphs of the sort shown in Figure 10 and Table 1 should prove to be useful in discussions with decision makers and the public about the types of progress that are (or are not) being made.

The right panel of Figure 10 demonstrates how estimates of slope or change between any two years of the flux record are highly dependent on the particular choice of starting or ending date for computing the change. This is also true for concentration records, but is generally less pronounced. Estimates of change based on the flow-normalized records will be much more stable over time and serve as a much more meaningful representation of progress toward water-quality goals. As an example: the estimated change in flux between 2002 and 2008 is an increase of 55%, but between 2003 and 2008, it is a decrease of 70% (because 2002 was a year of much lower flow than 2008 and 2003 was a year of much higher flow than 2008). In contrast, the estimated change in flow-normalized flux between 2002 and 2008 is a decrease of 1%, but between 2003 and 2008, it is a decrease of 4%. These latter expressions of change provide the public and decision makers with a much clearer and consistent perception of the rate of progress taking place in the watershed.

Having said all of this about the advantages of using these flow-normalized records to describe progress, it should not be forgotten that when the questions relate to the actual time-history of concentrations or fluxes, one should not use the flow-normalized records. The particular pattern of low- and high-flow months or years that has happened can be crucial to the ecological history of the river or a downstream receiving water body (such as Chesapeake Bay). The estimates of concentration or flux computed without flow-normalization are appropriate for many purposes such as: calibrating or testing models of processes that take place in the river or downstream water body, for assessing the threats to human health for those who come in contact with the water, or the treatment costs for waters withdrawn from the system and treated.

The flow-normalization method is only appropriate if we believe that the probability distribution of discharge for a given day of the year has not changed over the period of record. This would rule out its use if there have been substantial changes in the processes that govern streamflow in the watershed over the period of water-quality record. Examples of such changes could include: construction of a large dam upstream of the monitoring location, a substantial decline in groundwater levels that leads to a reduction in base flow, a substantial change in the consumptive use of water, or a substantial change in climate. If one or more of these had taken place upstream of the monitoring location during the period of water-quality record being studied, the flow-normalization method would not be appropriate. Determining if flow-normalization is appropriate should be viewed as a matter of judgment. The determination that a statistically significant trend exists in some aspect of the discharge record (e.g., trends in certain months or trends in low flows) is not a sufficient basis for rejecting the use of flow-normalization. The appropriate question to ask is whether the changes in discharge that have taken place during this period of record are likely to have a practical significance for the water-quality record of the watershed. Conducting formal hypothesis tests for stationarity is not appropriate for making this determination, given that it would require 365 tests, and certainly the null hypothesis would be rejected in some cases. At this point in the development of the WRTDS method this issue of nonstationarity of discharge is simply a caveat. The question that the user must pose to determine if the use of this simple flow-normalization procedure is appropriate is this: is there a strong basis for believing that the probability distribution of streamflow for any portion of the year has substantially changed between the beginning and end of the period of water-quality record and is this change large enough to be of practical significance? Development of formal procedures for the flow-normalization process in light of substantial nonstationarity of discharge would be an important future WRTDS enhancement.

It is important to provide a note of caution about interpretations of the flow-normalized records near either end of the time series. A smoothing procedure such as WRTDS will always produce less stable estimates near the ends than in the middle (this applies both to the actual estimates and the flow-normalized estimates). One should expect that after another year of data is collected, estimates for the last few years will be somewhat different than those that are computed today. This makes sense because we are using observations both before and after any given time of interest to help inform those estimates. Experiments have been run on several records to simulate changes due to the addition of new data. What these experiments show is modest changes in the trend magnitude of flow-normalized records as the first year or two are added to the record (a few percent), followed by very small changes (typically a percent or less) as a few more years are added to the record. In those cases where the underlying change in water quality is rather abrupt, the estimates at the end of the record may change by a larger amount, because WRTDS flow-normalized records are designed to provide a relatively smooth description of changes in the behavior of the system. When the changes are largely driven by changes in nonpoint sources or groundwater inputs or cumulative changes across a large number of point sources, this assumption of gradual change is likely to be appropriate. If the conditions were dominated by a single point source that underwent a major upgrade, then the WRTDS approach could have the effect of making a very abrupt change in water quality appear gradual. For the large watersheds for which these methods are designed, this potential shortcoming of the method is of limited consequences.

### A Second Example from the Chesapeake Bay Watershed, Nitrogen Concentrations in the Choptank River

**FIGURE 11:**
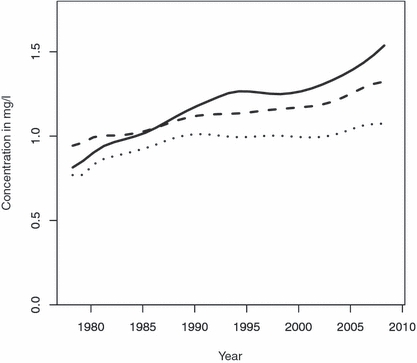
Estimated Concentration of Dissolved Nitrate Plus Nitrite, Choptank River Near Greensboro, Maryland, Evaluated at April 1 of Each Year for Discharge Values of 1.5 (solid line), 7 (dashed line), and 14 (dotted line) m^3^/s, Showing the Much Steeper Rise of Concentration at Low Flows Than at High Flows.

A second example to consider briefly is the dissolved nitrate plus nitrite record from the Choptank River at a streamgage near Greensboro, Maryland. It has characteristics quite different from the Patuxent River total phosphorus example. The Choptank basin has a low population density and a high intensity of agriculture. In this case, the nutrient of interest is nitrogen. It may be preferable to do this analysis for total nitrogen, but those records contain some censored values. Because the WRTDS method has yet to be implemented and tested for censored values, the analysis here will be for dissolved nitrate plus nitrite. (This extension of the method is a very high priority for future enhancements of WRTDS.) Unlike the Patuxent River site, there are no sewage treatment plants upstream. Also, in this situation, groundwater is an important source of the nutrient and it has been documented that groundwater nitrate concentrations have been increasing over the years 1988-2001 (Debrewer *et al.*, 2008). This dataset consists of 557 observations from 1979 through 2008. A good way to view the changes that have happened in the system is to use WRTDS to consider changes in concentration over the period at a particular time of year at three selected discharge values (similar to Figure 6). In this case, the date selected is April 1, and the three discharge values are 1.5 m^3^/s (near the 10th percentile discharge for this time of year), 7 m^3^/s (near the 75th percentile), and 14 m^3^/s (near the 90th percentile). These results are plotted in Figure 11. This figure shows that concentrations are consistently higher for lower discharges such as 1.5 or 7 m^3^/s, when compared with 14 m^3^/s, but in addition the trend in concentration at the lowest of these flows is much more pronounced than the trend at the higher discharges. For example, at a discharge of 1.5 m^3^/s, the increase from April 1, 1980, to April 1, 2006, is from 0.90 to 1.43 mg/l, an increase of 0.53 mg/l or 59%. For a discharge of 14 m^3^/s, the increase is from 0.83 to 1.06 mg/l, an increase of 0.23 mg/l or 28%. This suggests that much of the increase in nitrate plus nitrite in this stream comes from the groundwater discharge to the stream. There may also be increases in the concentrations that come in storm flow, but they are not as large (in absolute or relative terms) as the increases at low flow. The crossover in the curves for the two lower discharge values suggests that in the early years, at times of very low discharge, the base flow that made up most of streamflow was coming from deeper, less-contaminated parts of the aquifer, but over time these deeper parts of the flow system were becoming more affected by nitrate contamination from the surface. If these results are viewed from the perspective of a discharge *vs.* concentration relationship, we see that the shape of that relationship has changed over the 30-year period. In the early years, the slope of the concentration *vs.* discharge curve goes from positive to negative as discharge increases, but in the past 20 years the entire curve is downward sloping. This is another good example of the issue raised in the beginning of this paper, related to the second desired attribute of a new method: a highly flexible representation of the discharge *vs.* concentration relationship.

**FIGURE 12:**
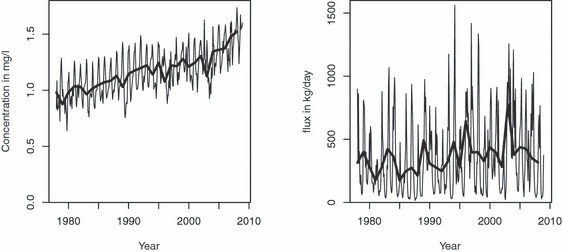
Left Panel Shows the Estimated Monthly (light line) and Annual (heavy line) Estimated Concentrations for Dissolved Nitrate Plus Nitrite, Choptank River Near Greensboro, Maryland. The right panel shows the monthly and annual estimated flux.

The results of computations of estimated concentrations and fluxes as monthly and annual averages are shown in Figure 12. The upward trend in concentration is quite clear, viewed either in the monthly or annual time series. Because concentrations are lower at times of higher discharge, two high flow years, 1996 and 2003, stand out as years of low concentration. The flux history is less clear because higher discharges, while having lower concentrations, carry higher fluxes. These same two wet years appear as positive spikes in the long-term flux record. Showing the monthly record can be useful in helping nontechnical audiences understand the very large range of variability of this system, with fluxes changing by as much as two orders of magnitude from month to month. These graphs also demonstrate how misleading short-term trends can be. From 2003 to 2008, this watershed has experienced progressively dryer conditions each year. (At the time when this analysis was being carried out, it was already clear that 2009 was a much wetter year than the years just before it. There is no reason to think that this six-year trend is more than a random occurrence). As a consequence, average concentrations as estimated by WRTDS have increased by 36% between 2003 and 2008. In contrast, the estimated flux has decreased by 59% over this same period. These contradictory results are not helpful to decision makers and the public who want to understand progress toward clean water goals. These changes are primarily artifacts of the particular pattern of streamflow over this period and tell us very little about progress toward the goals of reduced nutrient inputs to the Chesapeake Bay.

**FIGURE 13:**
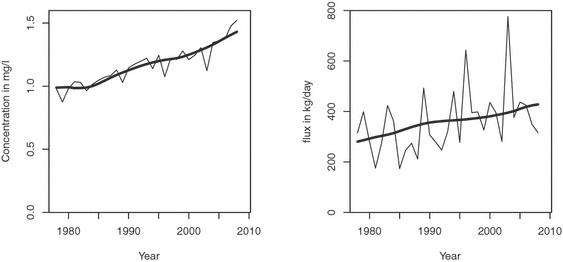
The Left Panel Is the Annual Average Estimated Dissolved Nitrate Plus Nitrite Concentration (light line) and the Flow-Normalized Annual Average Estimate (heavy line), for the Choptank Rver Near Greensboro, Maryland. The right panel contains flux estimates. In both cases, the annual estimates (light line) are the same as those plotted as the heavy line in the two panels of Figure 12.

Thus, the flow-normalized estimates shown in Figure 13 are quite useful. It shows the overall trends taking place, with the influence of random discharge-induced variations removed. Figure 13 shows that both concentration and flux have been increasing at a rather constant rate over the entire period and that the particular pattern of streamflow conditions over the 2003-2008 period have confounded the story, making the concentration trend appear much steeper than is realistically the case, and making the flux trend appear to be the opposite sign because of the particular sequence of discharges that went from very high to low over that period. From a flow-normalized perspective, concentrations rose by only about 10% over the years 2003-2008 and flux rose by about 8%. Table 2 shows the pattern of slope changes over different decades. These results suggest some degree of acceleration of the increase in the 2000 to 2008 period when compared with the 1990 to 2000 period. This is important information for decision makers concerned about progress on nitrogen control in this watershed. Water quality in terms of dissolved nitrate plus nitrite is deteriorating and appears to be doing so at an increasing rate. This is true whether it is evaluated in terms of concentrations or fluxes.

**TABLE 2:** Trend Slopes for Flow-Normalized Annual Average Values for Various Time Periods, Dissolved Nitrate Plus Nitrite, Choptank River Near Greensboro, Maryland.

	Concentration Results		Flux Results	
Time Span	Slope (mg/l per year)	Slope (% per year)	Slope (kg/day per year)	Slope (% per year)
1980-1990	+0.013	+1.4	+6.4	+2.2
1990-2000	+0.012	+1.1	+2.5	+0.7
2000-2008	+0.021	+1.8	+5.9	+1.6

### Application Across the Chesapeake Bay Watershed

One of the motivations for the type of analysis developed here is that it can be applied in a consistent manner across the many rivers that enter an estuary such as Chesapeake Bay, and can provide estimated time series (at time steps of days, months, years, or even decades) of fluxes from each of the several monitored rivers that enter the estuary. In the case of the Chesapeake Bay, the RIM network has consistently collected water-quality and discharge data for nine rivers over the roughly 30-year period from 1979 to 2009. The RIM sites sample water from about 78% of the land area of the Chesapeake Bay watershed (Langland *et al.*, 1995). The Patuxent and Choptank River datasets used above are both products of this network. The network monitoring sites are located as far downstream as possible, but above the head of tide, for the nine largest rivers draining to the Bay. They constitute an important part of the total inputs of nutrients to the Bay (based on the Chesapeake Bay Program Watershed model, Phase 4.3, they are currently estimated to be about 60% of total phosphorus and total nitrogen, source personal communication, Katie Foreman, Chesapeake Bay Program Office, January 2010). To determine the total inputs to the Bay, inputs from these rivers must be combined with inputs from the part of the watershed that is not monitored (both via surface-water inputs and via groundwater), from the point sources that are downstream of the monitoring sites, and from the atmospheric inputs directly to the bay.

The RIM datasets were all analyzed using WRTDS, with the same half-window widths in all cases (time, 10 years; discharge, 2 natural log units; and season, 0.5 year). All of these sites had an average of 15 to 24 samples per year on average. If the analysis was being conducted on a set of sites with substantially different sampling densities, it might have been appropriate to vary the windows across sites. The analyses include both total phosphorus and dissolved nitrate plus nitrite, both flux and concentration, and both annual estimates and flow-normalized estimates. This paper will not explore the other components of the inputs to the bay and makes no attempt to compute trends in total inputs. However, we believe that the results are useful to describe patterns of long-term change for individual tributaries and to make comparisons across the tributaries.

Table 3 names the RIM sites, showing their drainage areas, land use, and amount of major wastewater discharges upstream. A map of these sites and their upstream watersheds is shown in Appendix S2 of the Supporting Information.

**TABLE 3:** River Input Monitoring Program Data Collection Sites, With Information About Drainage Area, Land Use, and Wastewater Discharges (U.S. Geological Survey, 2010, http://va.water.usgs.gov/chesbay/RIMP/generalinfo.html).

		Land Use (%)				
Station Name	Upstream Land Surface Area (km^2^)	Urban	Agricultural	Forested	Other	Major Upstream Wastewater Discharges (m^3^/day)
Susquehanna River near Conowingo, Maryland	70,200	2	29	67	2	1,650,000
Potomac River at Chain Bridge, Washington, D.C.	30,000	3	35	61	1	477,000
James River at Cartersville, Virginia	16,200	1	16	80	3	338,000
Rappahannock River near Fredericksburg, Virginia	4,130	1	36	61	2	17,800
Appomattox River at Matoaca, Virginia	3,480	1	20	72	7	4,200
Pamunkey River near Hanover, Virginia	2,790	1	24	68	7	18,900
Mattaponi near Beulahville, Virginia	1,560	1	19	69	11	400
Patuxent River at Bowie, Maryland	901	13	41	38	8	114,000
Choptank River near Greensboro, Maryland	293	1	50	29	20	0.0

Land-use data from Vogelmann *et al.* (1998).

**FIGURE 14:**
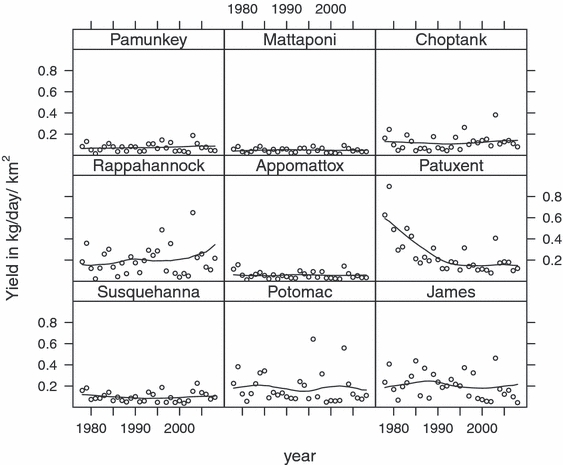
Total Phosphorus Yields for the Nine River Input Monitoring Sites Using WRTDS Method. Circles are the average annual estimates. The line represents the flow-normalized annual estimates.

Figure 14 aggregates the analyses of total phosphorus fluxes across all of the RIM sites into a single graphic, showing the annual average estimates and the flow-normalized annual average estimates for all of the sites. Given that the sizes of the nine watersheds range over more than two orders of magnitude, for purposes of comparison, the fluxes are recalculated as yields (yield is flux per unit drainage area) expressed as kg/day/km^2^ and all nine watersheds are shown at the same scale. Four of the watersheds shown in Figure 14 have much lower yields than the others (Pamunkey, Mattaponi, Appomattox, and Susquehanna). The first three of these drain almost entirely Coastal Plain and Piedmont watersheds and thus have low stream gradients throughout their length. The Susquehanna is sampled at the downstream end of a series of reservoirs where substantial amounts of sediment and associated phosphorus are deposited. The Patuxent record as discussed above shows the very substantial decrease in phosphorus flux over the 31-year record. Five of the watersheds had positive slopes for the whole period, and three others had negative slopes but of a much lower magnitude than the Patuxent. From a total bay watershed perspective it is important to note that the two largest rivers (Susquehanna and Potomac) both had long-term decreases in flux. Three sites show increases during the 2000-2008 period of more than about 1.5% per year: the Rappahannock (8.4% per year), the James (+2.5% per year), and the Choptank (+1.9% per year). All of the others are showing either small increases or small decreases during the most recent eight-year period. Table 4 summarizes these results for total phosphorus fluxes, expressed both as rates of change in percentage terms as well as absolute changes in terms of actual fluxes. Similar results for total phosphorus concentration (graphical and tabular) are shown in Appendix S2 of the Supporting Information.

**TABLE 4:** Changes in Total Phosphorus Flux for the Nine RIM Sites for Two Periods: 1978-2008 and 2000-2008.

	1978-2008		2000-2008	
River	Slope (% per year)	Flux Change (kg/day)	Slope (% per year)	Flux Change (kg/day)
Susquehanna	−0.4	−990	+1.9	+970
Potomac	−0.3	−530	−2.0	−940
James	+0.5	+480	+2.5	+590
Rappahannock	+4.0	+780	+8.4	+580
Appomattox	−0.2	−10	+0.8	+12
Patuxent	−2.5	−400	+0.2	+2
Pamunkey	+1.2	+64	+1.1	+19
Mattaponi	+0.7	+12	+0.1	+0
Choptank	+0.3	+3	+1.9	+5

Flux change is the flow-normalized annual flux estimate at the end of the period minus the flow-normalized annual flux estimate at the beginning of the period. The slope is this flux change per year expressed in percentage terms over the period.

**FIGURE 15:**
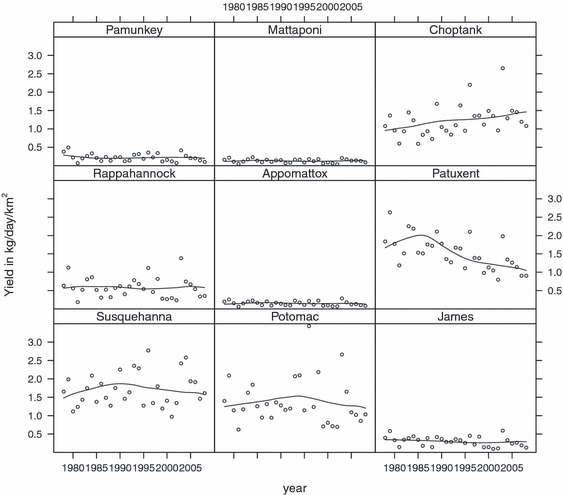
Dissolved Nitrate Plus Nitrite Yields for the Nine River Input Monitoring Sites Using WRTDS Method. Circles are the average annual estimates. The line represents the flow-normalized annual estimates.

Figure 15 presents the same type of results for dissolved nitrate plus nitrite. For nitrate plus nitrite four sites stand out as having consistently lower yields: Mattaponi, Pamunkey, Appomatox, and James. Over the full period of record only two of them show substantial relative rates of change: the Choptank River (+1.8% per year) and the Patuxent River (−1.2% per year). The Choptank nitrogen trends were discussed above. The Patuxent nitrogen trends are likely a result of the investments made in advanced wastewater treatment in the watershed. Over the full period the percentage changes in the two largest rivers were small, +0.2% per year for the Susquehanna and −0.1% per year for the Potomac, but these represent the largest absolute changes in flux of any of the rivers. Over the shorter more-recent period of 2000-2008, two sites show annual rates of increasing flux of >1% per year (Choptank and James) and four sites show decreases of >1% per year (Patuxent, Potomac, Pamunkey, and Appomatox). This recent period shows rather substantial declines in the two largest rivers and these translate into large reductions in the total river inputs of nitrate plus nitrite to the bay.

Tabular results for dissolved nitrate plus nitrite fluxes are given in Table 5. Similar graphs and tables are shown for concentration histories in Appendix S2. Appendix S3 contains tables of the complete set of results for both total phosphorus and dissolved nitrate plus nitrite, for both flux and concentration, and both annual estimates and flow-normalized annual estimates. Using these tables, amounts of change and rates of change can be computed for any time span covering the 31-year period.

**TABLE 5:** Changes in Dissolved Nitrate Plus Nitrite Flux for the Nine RIM Sites for Two Periods: 1978-2008 and 2000-2008.

	1978-2008		2000-2008	
River	Slope (% per year)	Flux Change (kg/day)	Slope (% per year)	Flux Change (kg/day)
Susquehanna	+0.2	+6,400	−0.9	−8,600
Potomac	−0.1	−1,200	−1.5	−4,800
James	−0.5	−880	+1.2	+400
Rappahannock	+0.1	+69	+0.4	+71
Appomattox	+0.2	+21	−1.3	−55
Patuxent	−1.2	−560	−1.9	−160
Pamunkey	−1.0	−240	−1.4	−73
Mattaponi	+0.3	+17	+0.7	+10
Choptank	+1.8	+147	+1.6	+48

Flux change is the flow-normalized annual flux estimate at the end of the period minus the flow-normalized annual flux estimate at the beginning of the period. The slope is this flux change per year expressed in percentage terms over the period.

The results presented for the RIM network are intended to be indicative of the kinds of representations of long-term variations and trends that are possible with the WRTDS method. It is not the goal of this paper to explore explanations of the patterns revealed in the analysis. However, it is the goal of this paper to demonstrate the rich set of questions that the analysis can raise. As mentioned in the seventh item of “Seven desired attributes of a new approach for the analysis of long-term water-quality data,” the outputs are intended to be useful not only as measures of relative success for achievement of water-quality goals but also as a gateway to diagnostic analysis of the nature of and possible reasons for the changes that are being observed. These results can serve as a starting place for more detailed explorations of concentration and flux histories focused on various times of year and/or various flow conditions. These results can also be related to information about the history of changes in the watershed (population, point-source loadings, fertilizer applications, land use change, and many other topics). The WRTDS approach provides a variety of tools to use in a diagnostic manner to better describe and understand the changes taking place and thus to help guide future nutrient-control strategies.

## Suggested Topics for Exploration and Future Improvements in WRTDS

This paper is intended to introduce a new approach to water-quality trend analysis. In the course of its development and through discussions with many experienced practitioners, we see a set of methodological questions and extensions that should be the subject of ongoing or future development. The following is a list of suggested topics in need of future exploration or improvements in WRTDS.

1. At present, WRTDS does not have the capability of analyzing censored data (records where some observations are recorded as “less than” the limit of detection). Conceptually, this is an improvement that can readily be made using censored regression approaches. There would certainly be questions about the degree of censoring that the method can tolerate and questions of statistical robustness, but it should be possible to make this improvement.
2. This approach considers only three factors influencing concentration: time, time of year, and the discharge on the day of sampling. There is good reason to consider the history of discharge (at time scales of days to years) as explanatory variables (see, e.g., Hirsch, 1988; Evans and Davies, 1998; Vecchia, 2005; Vecchia *et al.*, 2009). Concentrations are influenced not just by the discharge at the time of sampling, but by whether discharge is rising or falling, or whether the watershed has experienced weeks or months of particularly dry or wet conditions. Adding these factors to the analysis may prove useful for increasing the accuracy of the method, although it will add complexity and potential model error.
3. As mentioned above, the flow-normalization method is built on an assumption that streamflow is stationary (for any given time of year). There is a need to generalize the flow-normalization method to consider the role of changing streamflow conditions due to factors such as reservoir storage, groundwater declines, or climate change.
4. In addition to estimates of averages of concentrations and fluxes, it would be useful to have the method report out temporally varying estimates of the probability of exceeding some threshold (based on considerations of human or ecosystem health). The results of the regressions could be expressed, not in terms of an expected value, as is done here, but in terms of an estimated probability that on any given day the concentration would have exceeded a specific threshold (e.g., a water-quality standard). The analysis could then report out estimates of long-term history of the frequency with which those thresholds have been exceeded.
5. Another improvement in the WRTDS system would be the simultaneous consideration of multiple water-quality variables. Particularly, where the goal is diagnostic analysis of water-quality conditions, it is useful to consider questions such as the relative amounts of different forms of a particular element (e.g., nitrate, nitrite, ammonia, total nitrogen, etc.). The tendency for some forms to move with particles, others to move in dissolved form, some to move through groundwater, and some to react and change form in transport, can be exploited to help understand source, transport, and fate. These multivariate approaches may prove to be useful.
6. The method as presently implemented has no objective rules for setting the window widths for the smoothing. By working with larger and more complete datasets that can be subsampled, it may be possible to optimize the selection of window widths.
7. Finally, the subject of error analysis is a crucial one to consider. Similar to the error analysis work by Gilroy *et al.* (1990), for a regression-based approach, there is a need for a method that can be used to estimate the uncertainty of estimates for individual months or years. The WRTDS results do not provide a “standard error” for any of the estimates made, and do not provide a means for conducting hypothesis tests or stating that a trend is so many percent per year, plus or minus some amount of uncertainty. Although the addition of uncertainty estimates would be desirable, it is less problematic when one views the analysis as a broadly descriptive and diagnostic function rather than a hypothesis testing function. Our underlying philosophy in this work is that water-quality change is a given, the task at hand is to provide a meaningful description of the nature and magnitude of the change.

## Conclusion

This paper is intended to open up the discussion of water-quality trends to new approaches that strive to extract the greatest amount of information from the data collected and to provide the greatest possible insight to inform policy. The WRTDS method is presented as a very specific, formal implementation of a new philosophy about water-quality data analysis. At this stage of the development, experimentation and improvement on this method is strongly encouraged. What is imperative is that water-quality professionals strive to use the rich datasets that are now available to extract the greatest amount of information from the data and communicate it to decision makers and the public.

## SUPPORTING INFORMATION

Appendix S1. Uncertainty analysis.

Appendix S2. Concentration results for the nine River Input Monitoring sites.

Appendix S3. The tables in this appendix provide estimates of concentration in mg/l, and flux (in both lbs/day and kg/day) for all sites, for total phosphorus and for dissolved nitrate plus nitrite, showing annual estimates and flow-normalized annual estimates.

Please note: Neither AWRA nor Wiley-Blackwell is responsible for the content or functionality of any supporting materials supplied by the authors. Any queries (other than missing material) should be directed to the corresponding author for the article.

[Correction after Online publication September 7, 2010: Figure 13c axis unit names changed.]
