# Supplementary material for: Weighted Regressions on Time, Discharge, and Season (WRTDS), with an Application to Chesapeake Bay River Inputs
Source: J Am Water Resour Assoc. 2010 Oct;46(5):857–80. doi: 10.1111/j.1752-1688.2010.00482.x (PMC3307614; doi:10.1111/j.1752-1688.2010.00482.x)
Supplement: Supplementary file 1 [file jawr0046-0857-SD1.doc]

Appendix A: Uncertainty analysis

Residuals of WRTDS estimates were computed and compared with simpler and more traditional approaches to trend analysis. The approach considers a range of six different models. These are shown in table A1. They proceed from the simplest (model 1) to the most complex (WRTDS which is model 6). Table A2 identifies these same models in words rather than equations.

| Model number | Expressed as an equation |
| --- | --- |
| 1 |  |
| 2 |  |
| 3 |  |
| 4 |  |
| 5 |  |
| 6 | WRTDS – same as 4 but coefficients vary |

Table A1: The six alternative models, stated as equations (terms defined in body of the paper)

| Model number | Model expressed in words |
| --- | --- |
| 1 | constant |
| 2 | linear trend in time |
| 3 | quadradic trend in time |
| 4 | linear in time and ln(Q) with seasonal terms |
| 5 | quadratic in time and ln(Q) with seasonal terms |
| 6 | WRTDS |

Table A2: The six alternative models, stated in words

The results for total phosphorus concentrations at the Patuxent River near Bowie, MD and the results for dissolved nitrate plus nitrite concentrations at the Choptank River, near Greensboro, MD are shown in table A3. These changes over the range of models are summarized in Figure A1, which shows the increasing fraction of the variance explained across the six models. For the Patuxent River data set, because of the very large trend, the linear trend model (model 2) was a large improvement over the no trend model (model 1), but because of the curvature of the trend the quadratic trend (model 3) resulted in still more improvement. Adding both the quadratic discharge and the seasonal terms (model 5) to the quadratic trend model results in still more improvement. But, the greatest reduction in variance was with WRTDS. The fact that WRTDS was substantially better than model 5 indicates that there are features of the data set that are not well fitted even by this 6 variable model. In the case of the Choptank data set, the time trend is less pronounced and is rather linear. Without the discharge and seasonal terms there is very little variance explained. It is also clear that adding a quadratic trend in time (going from model 2 to model 3 or model 4 to model 5) resulted in very minimal improvements, but the flexibility of model 6 resulted in a substantial improvement, similar to the improvement seen in the Patuxent example.

|  | Patuxent River near Bowie,  Total phosphorus | | Choptank River near Greensboro, Dissolved nitrate plus nitrite | |
| --- | --- | --- | --- | --- |
| Model | Variance  Explained | Percent RMSE | Variance  Explained | Percent RMSE |
| 1 | 0 % | 88 % | 0 % | 38 % |
| 2 | 23 % | 75 % | 5 % | 37 % |
| 3 | 34 % | 68 % | 5 % | 37 % |
| 4 | 35 % | 67 % | 39 % | 29 % |
| 5 | 47 % | 60 % | 43 % | 28 % |
| 6 | 56 % | 53 % | 56 % | 25 % |

Table A3: Percentage of variance explained for the log of concentration each of the 6 models is the variance explained relative to model 1, and percent root mean square error (Percent RMSE) of the residuals (in real space) using the Patuxent River near Bowie Maryland total phosphorus data (773 observations) and the Choptank River near Greensboro Maryland dissolved nitrate plus nitrite data (557 observations).


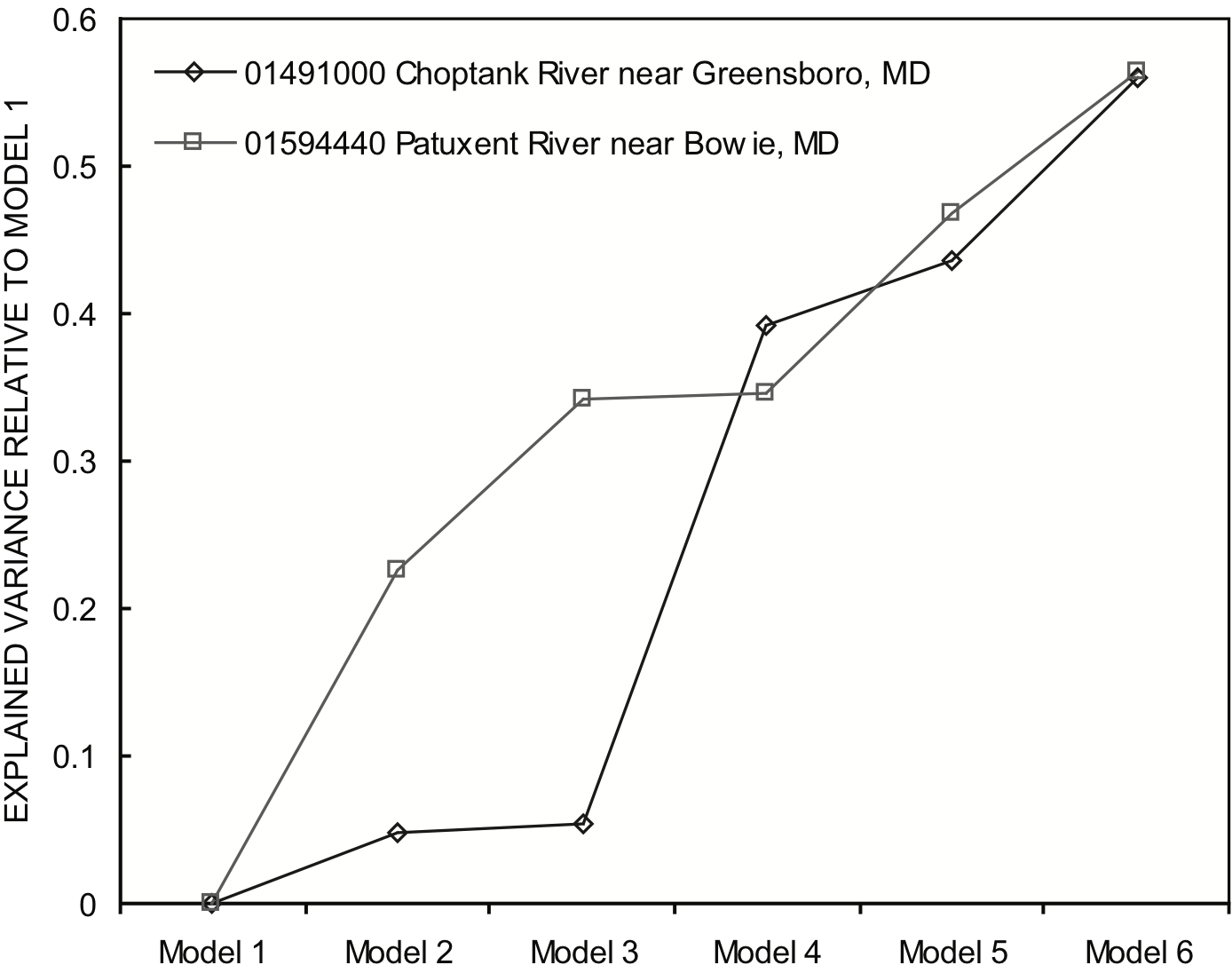


Figure A1: R-squared values for each of the six models, for Patuxent River total phosphorus data and Choptank River dissolved nitrate plus nitrite data.

One way to look at the fit of the various models is to view boxplots of their residuals. These are presented for the Patuxent in Figure A2 and for the Choptank in Figure A3.


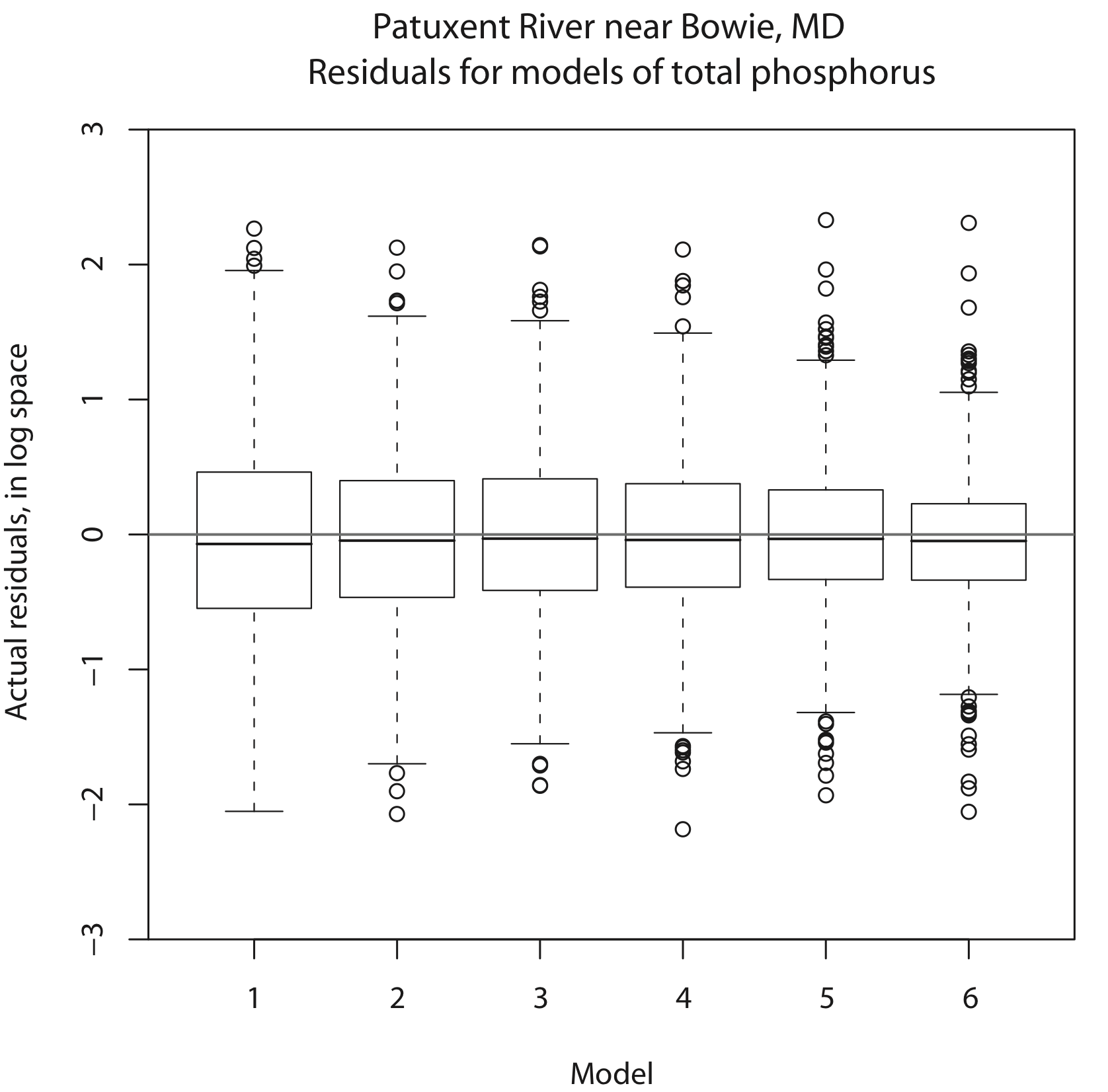


Figure A2: Log residuals, by model, Patuxent River total phosphorus data.


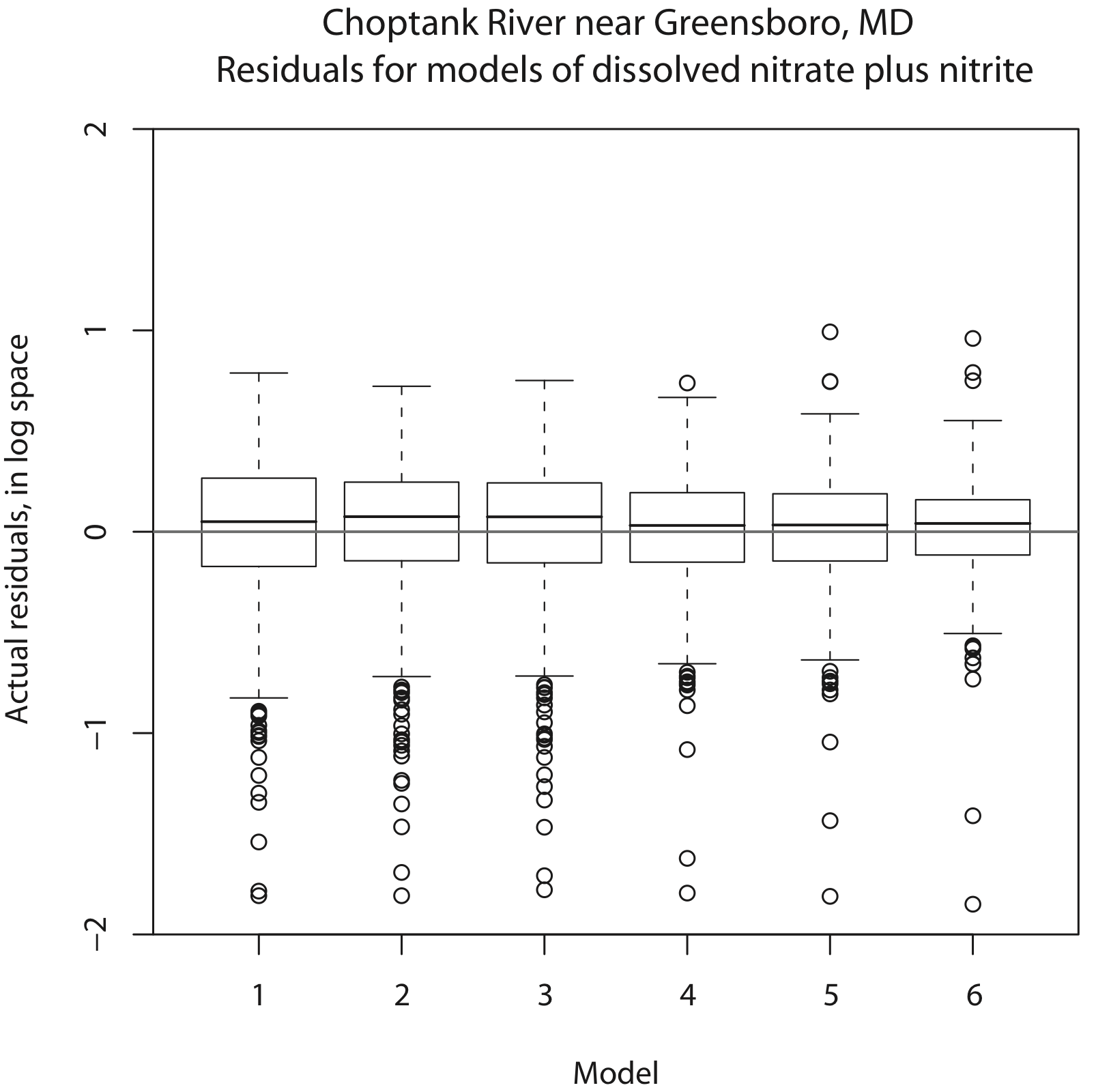


Figure A3: Log residuals, by model, Choptank River, dissolved nitrate plus nitrite data.

Both of these figures illustrate that the residuals tend to be rather symmetrical in log space, suggesting that any kind of model fitting is best done in log space. If the model fitting had been done in real space then the residuals would be highly skewed and thus coefficient estimates would be very prone to large variations from a few extreme observations. The figures also show that the distributions of the residuals narrows from model 1 to model 6, particularly when viewed from the interquartile ranges (the distance between the top and bottom of each box). It also reveals that both data sets have a modest number of observations that are quite extreme. For example, with the Choptank River nitrate plus nitrite data using model 6, half of the observations are in the range from about 78% to 128% of the estimated value, there is one value that is only 15% of the estimate (on the low side) and one that is as much as 270% of the estimate (on the high side). Users need to be mindful that while these models explain a non-trivial portion of the total variation in the data, there are instances when the system presents large “surprises.” These extremes should not be ignored, but neither should summary results be too highly effected by them, unless the surprise becomes a regular happening, in which case the model needs to adjust to the new reality. It is this adjustability that is an important feature to WRTDS. The windowing process assures that important changes in behavior do not have major effects far backward into the estimated history of the system, but when they begin to happen with some regularity, the model needs to adjust to them.

Another way to look at the quality of the different models is through plots of the observed concentrations versus the estimates derived from the model. Because the fitting was done in log space these graphs are created as log-log plots. Perfect agreement between the observed and the estimate follows the diagonal line in each plot. Figures A4 and A5 provide such a perspective for the same two cases.


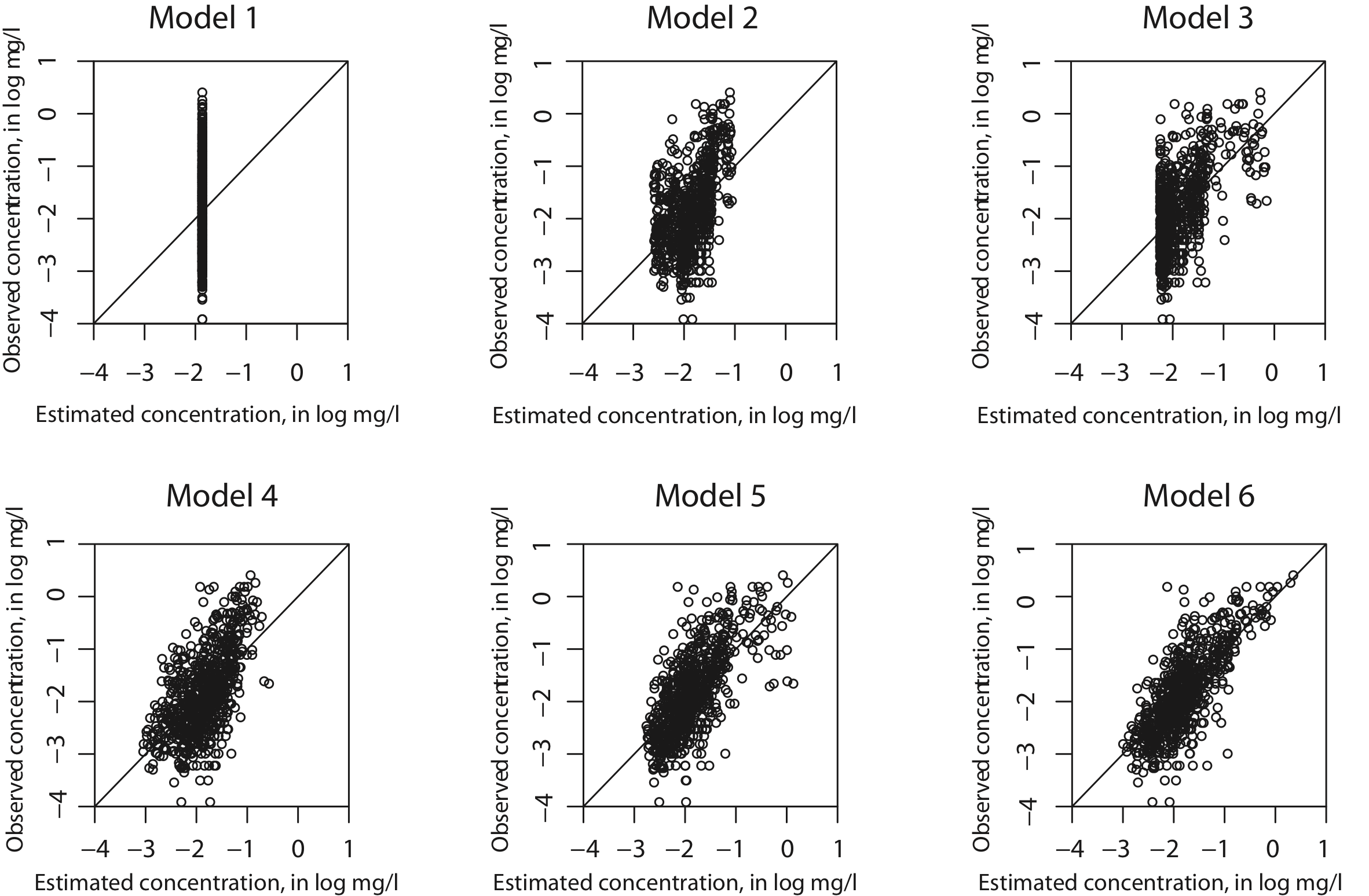


Figure A4: Observed values versus estimated values of log(concentration) for total phosphorus, Patuxent River near Bowie, MD, for each of the six models. Solid line is the line of perfect agreement.


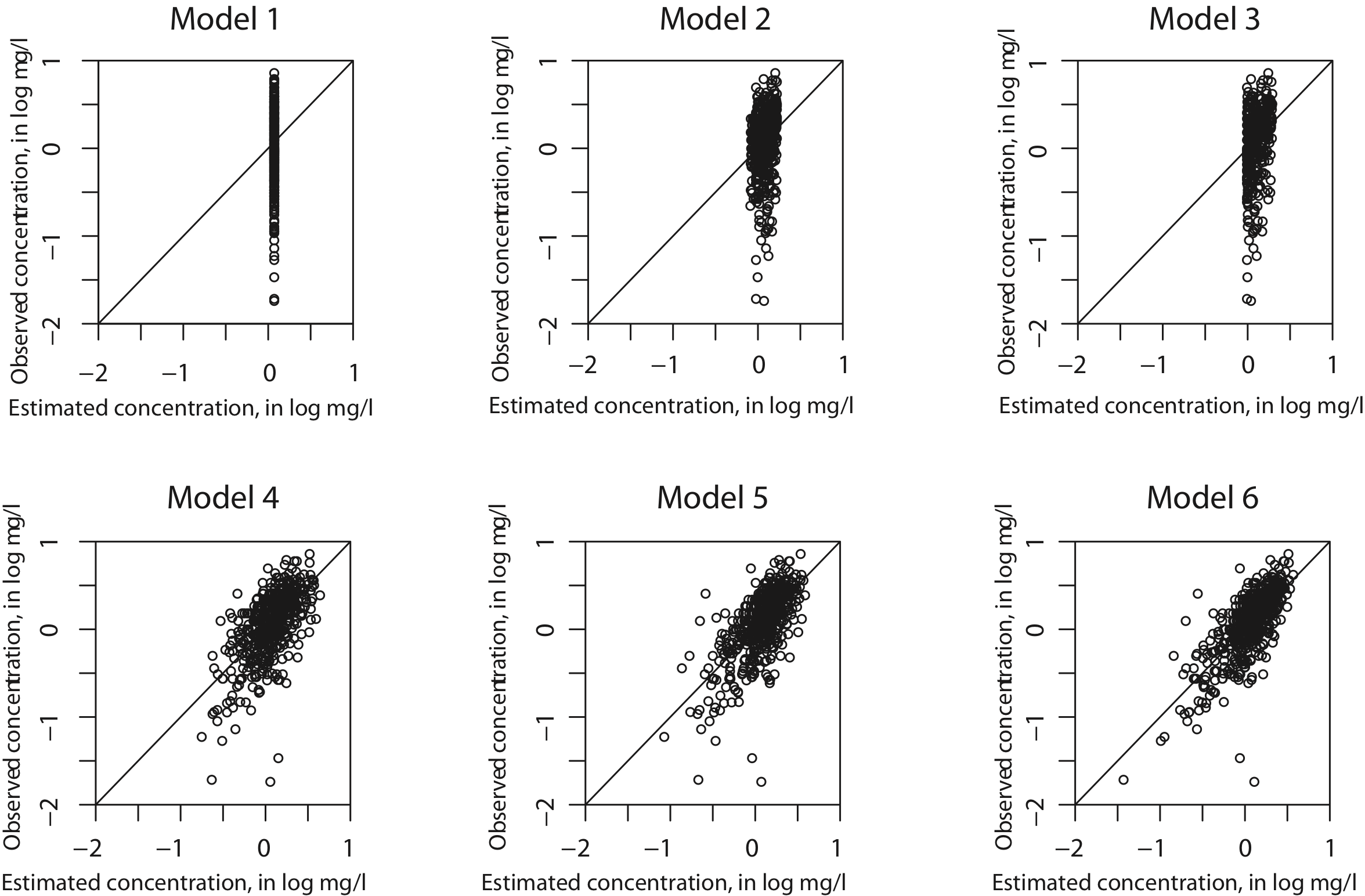


Figure A5: Observed values versus estimated values of log(concentration) for dissolved nitrate plus nitrite, Choptank River near Greensboro, MD, for each of the six models. Solid line is the line of perfect agreement.

Both figures show the progressive improvement of the fit of the model to the observed data. Nevertheless, for all models there is a tendency for the model estimates to be less variable than the actual observations (the model underpredicts high values and overpredicts low values). This result is to be expected by the nature of regression. Model 1, illustrates the case where the model is just a constant and none of the variance is explained. If there were a perfect model, explaining 100% of the variation, then the data would lie exactly on the 1 to 1 line.

Yet another way to look at the performance of the models is to consider the residuals as a function of time. The pattern we would like to see is that the cloud of data, at all times through the history of the record lies symmetrically around the horizontal zero line. This would indicate that there are no major swings in the data that are not captured by the model. These are depicted in Figures A6 and A7.


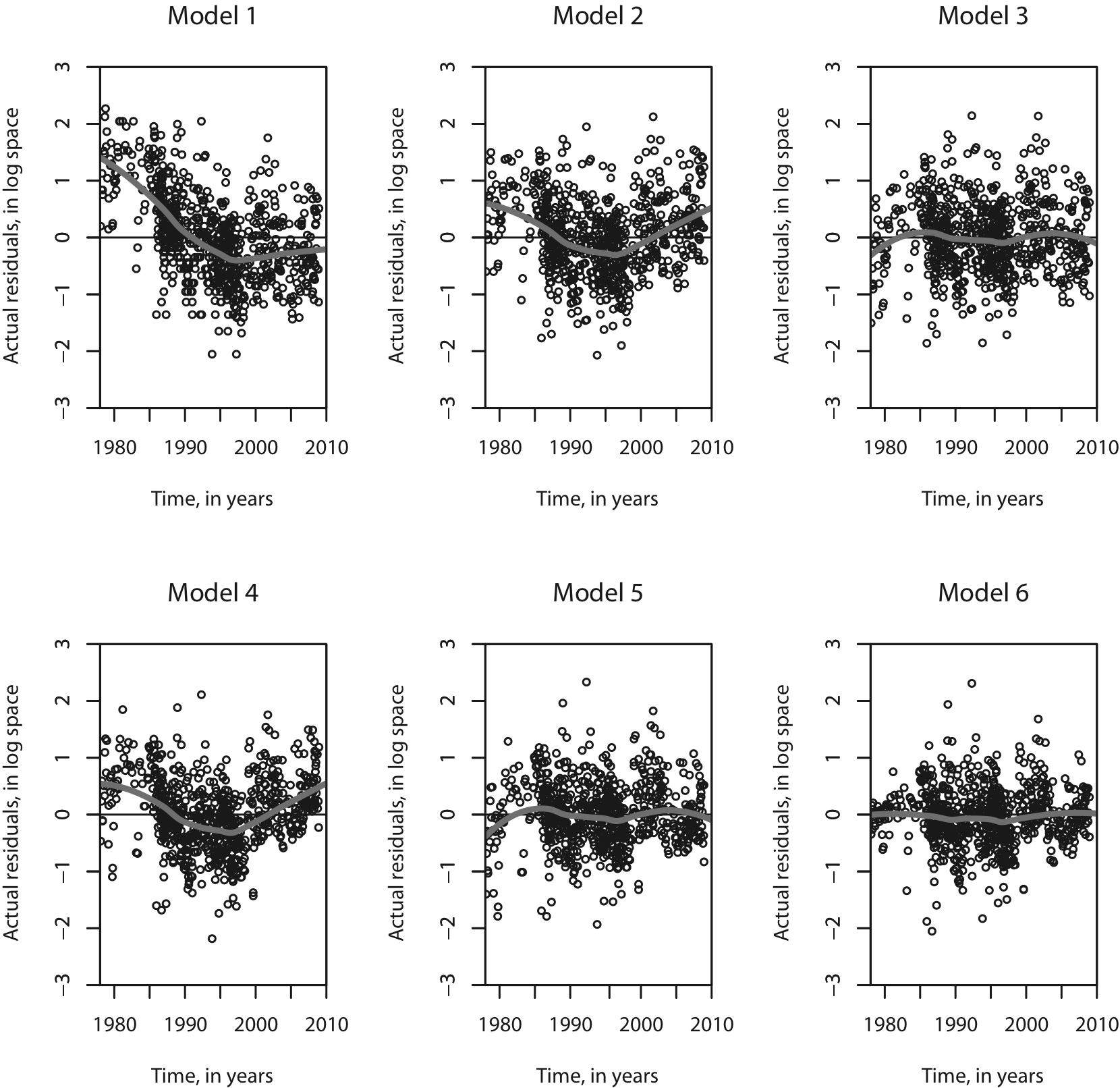


Figure A6: Residuals (in log space) of each of the six models, versus time, for total phosphorus, Patuxent River, near Bowie, MD. Heavy curve is a loess fit of the residuals over time.


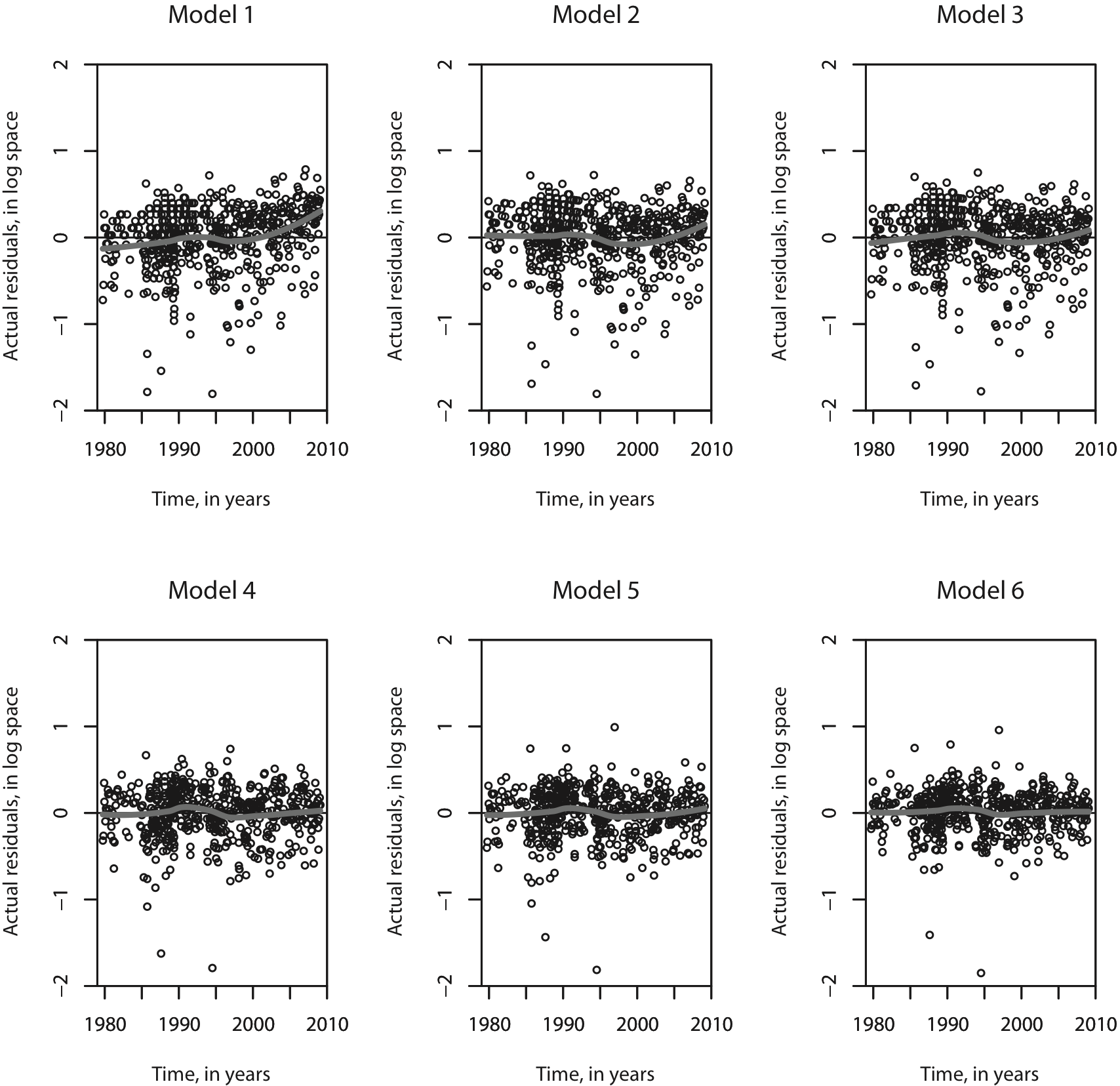


Figure A7: Residuals (in log space) of each of the six models, versus time, for dissolved nitrate plus nitrite, Choptank River, near Greensboro, MD. Heavy curve is a loess fit of the residuals over time.

For the Patuxent River, model 1, which has no time term has residuals that express the very substantial trend that exists in the data. Model 2, with its linear time term shows the poor fit because the curvature is not considered. Model 3, with the quadratic time trend, shows a reasonable temporal tracking of the trend, but is very noisy. Some improvements are made by model 4 and 5 because they use more information (flow and season) but both models fail to remove portions of the complex time trend in the data. Model 6 appears to remove much of the temporal signature of the trend, except for patterns that happen more rapidly, such as two or three years duration. In the case of the Choptank, the addition of a time trend term in model 2 brings about a small improvement, but the quadratic time trend in model 3 makes very little difference. The addition of flow and seasonal terms in model 4 make a substantial difference, making the residuals much closer to the zero line. Model 5 is somewhat better than 4 and model 6 is, in turn, better than model 5. The quadratic trend term used in models 3 and 5 makes little difference in fit.

Lastly, we can examine monthly box plots of the residuals for each model to determine if there any lingering seasonal pattern (figures A8 and A9). If a seasonal pattern was present in the residuals of a particular model, this would indicate that the model was unable to explain the seasonal variability and the residuals in a given month will exhibit bias. Models 1 through 3, which do not consider a seasonal component show a season a weak season pattern in the residuals; this occurs in the summer months for the Patuxent River (figure A8) and in the spring months for the Choptank (figure A9). When seasonal explanatory variables are included in the regression models (models 4 through 6), there is no observable seasonal pattern remaining in residuals for the Patuxent River site; however, the Choptank still appears to contain some seasonal pattern the residuals even for regression models that include seasonal explanatory variables. Nonetheless, it is evident from figures A8 and A9 that the WRTDS model contains the least amount of seasonal variation in the residuals and, as was observed in other plots of the residuals, the WRTDS model consistently resulted in the smallest residual values across all months.

These results, and many others explored in the process of developing and testing the WRTDS model suggest that it performs well over a wide range of cases. The data still contain large amounts of unexplained variation, and it is for that very reason that there is need for caution about the use of any trend estimation method that will rely on a few new observations to bring about an interpretation of an underlying change in the behavior of the system. It is only when, over a period of a few years, there are repeated observations that depart from historical behavior that the trend estimation model should begin to move substantially in the direction indicated by the recent observations.


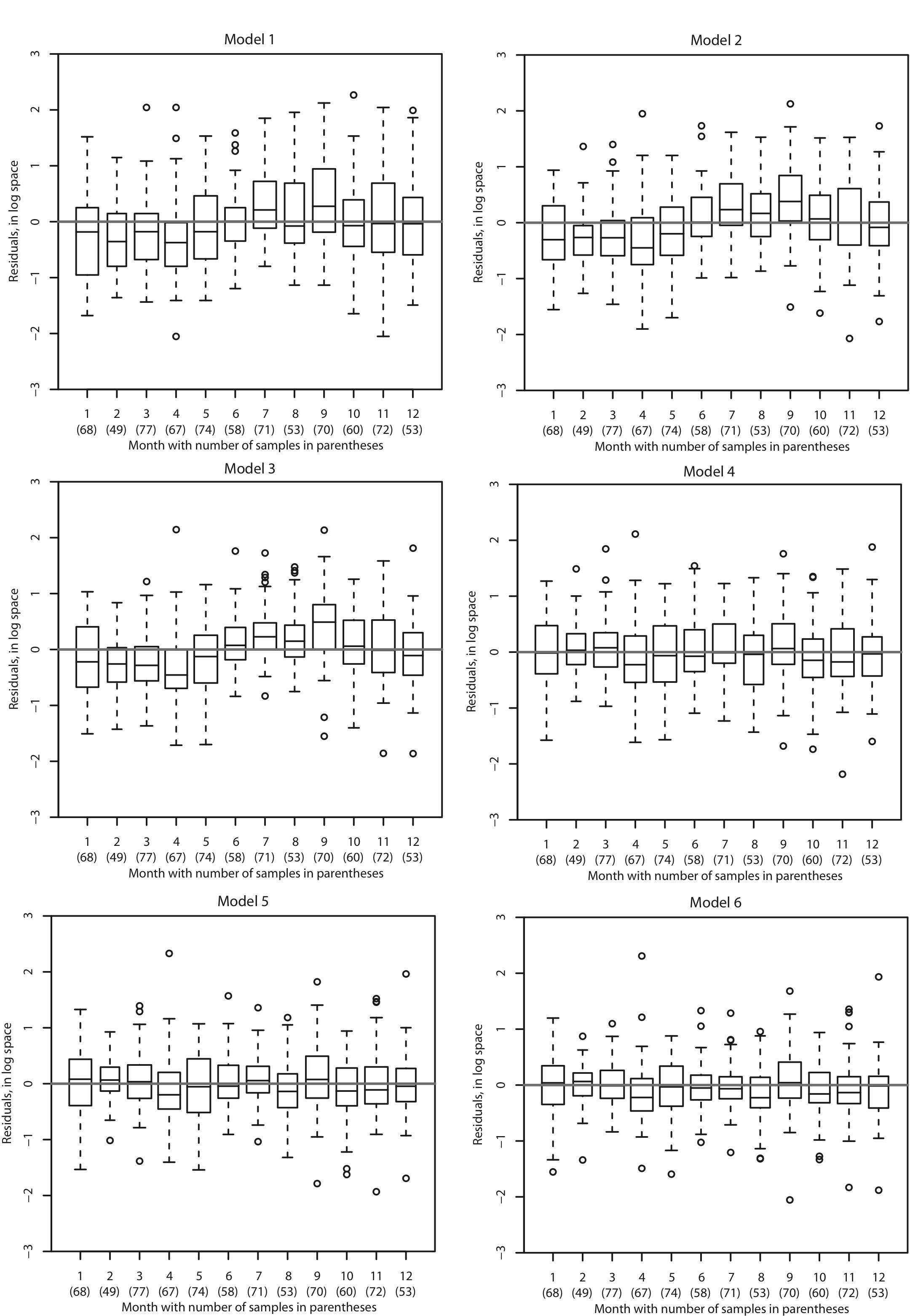


Figure A8. Monthly box plots of residuals (in log space) for each of the six models for total phosphorus, Patuxent River, near Bowie, MD.


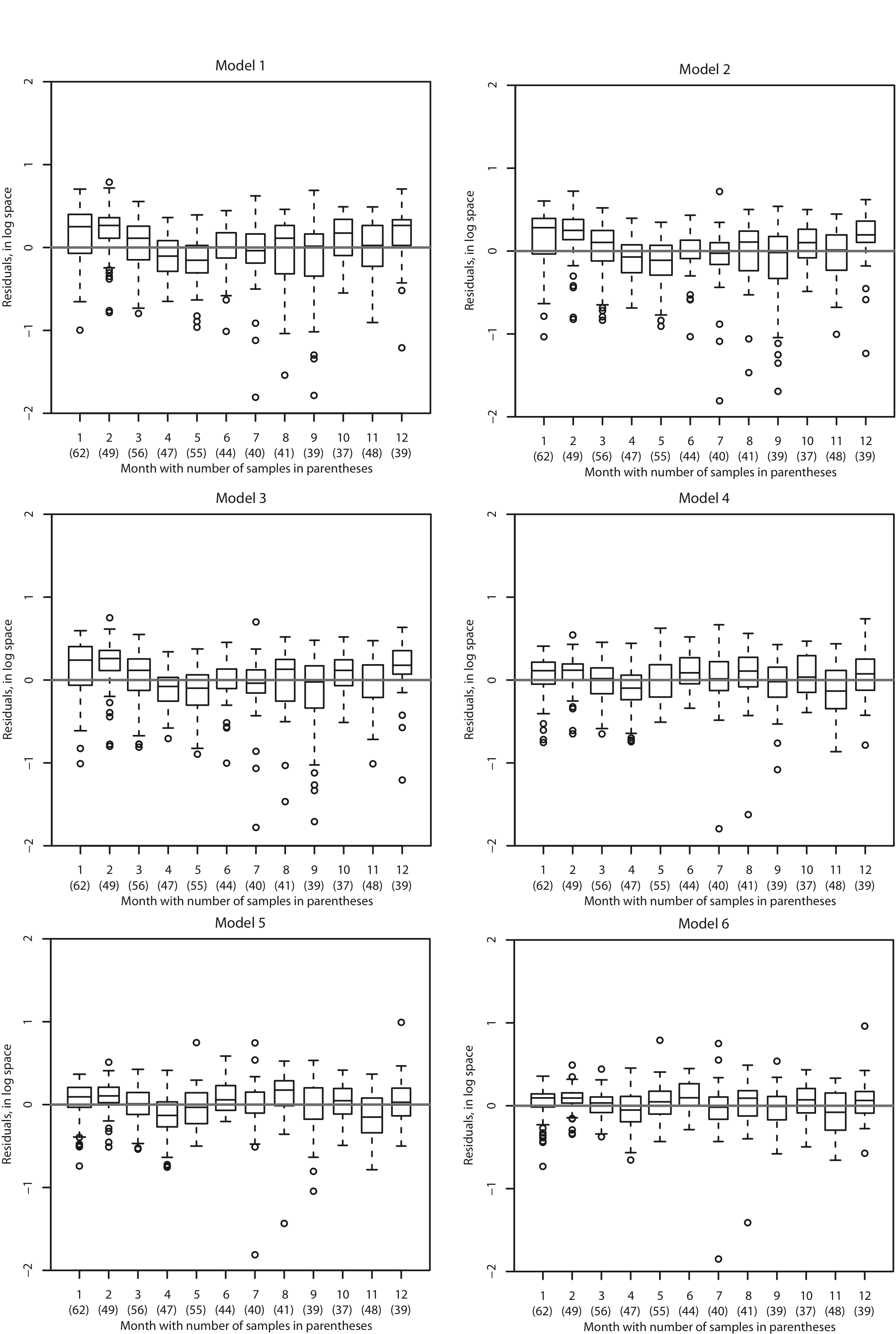


Figure A9. Monthly box plots of residuals (in log space) for each of the six models for dissolved nitrate plus nitrite, Choptank River, near Greensboro, MD.
