# Supplementary material for: Weighted Regressions on Time, Discharge, and Season (WRTDS), with an Application to Chesapeake Bay River Inputs
Source: J Am Water Resour Assoc. 2010 Oct;46(5):857–80. doi: 10.1111/j.1752-1688.2010.00482.x (PMC3307614; doi:10.1111/j.1752-1688.2010.00482.x)
Supplement: Supplementary file 2 [file jawr0046-0857-SD2.doc]

Appendix B: Concentration results for the 9 River Input Monitoring sites.


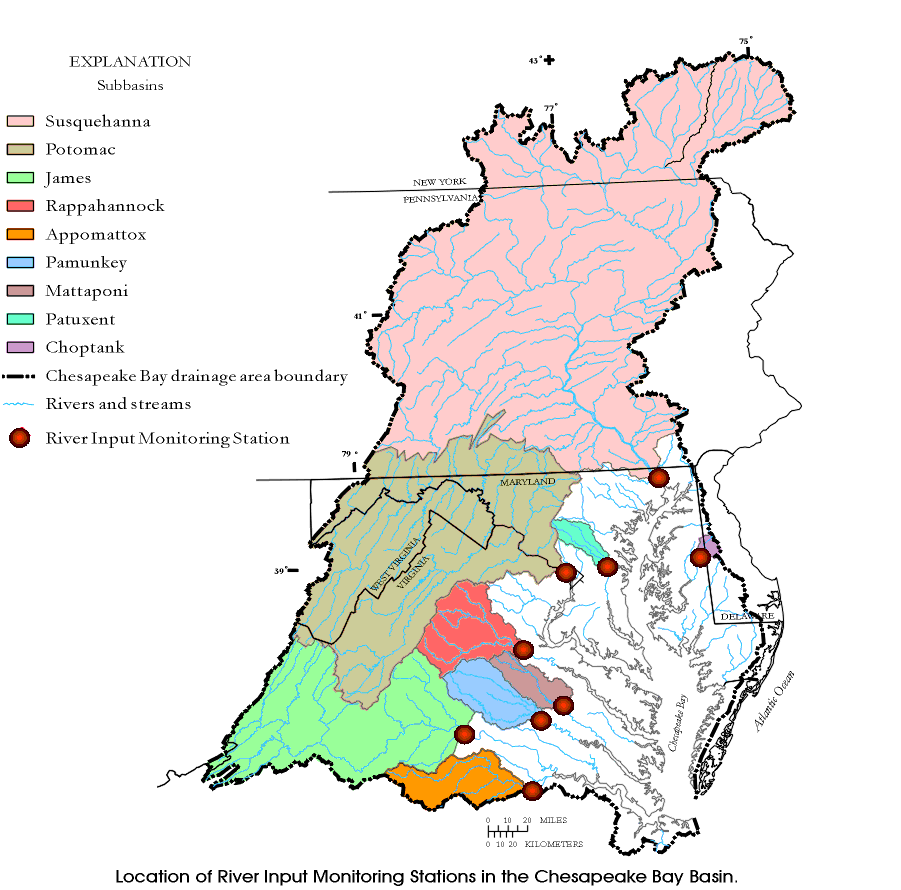


Figure B1 shows the location of the 9 River Input Monitoring stations and their watersheds.


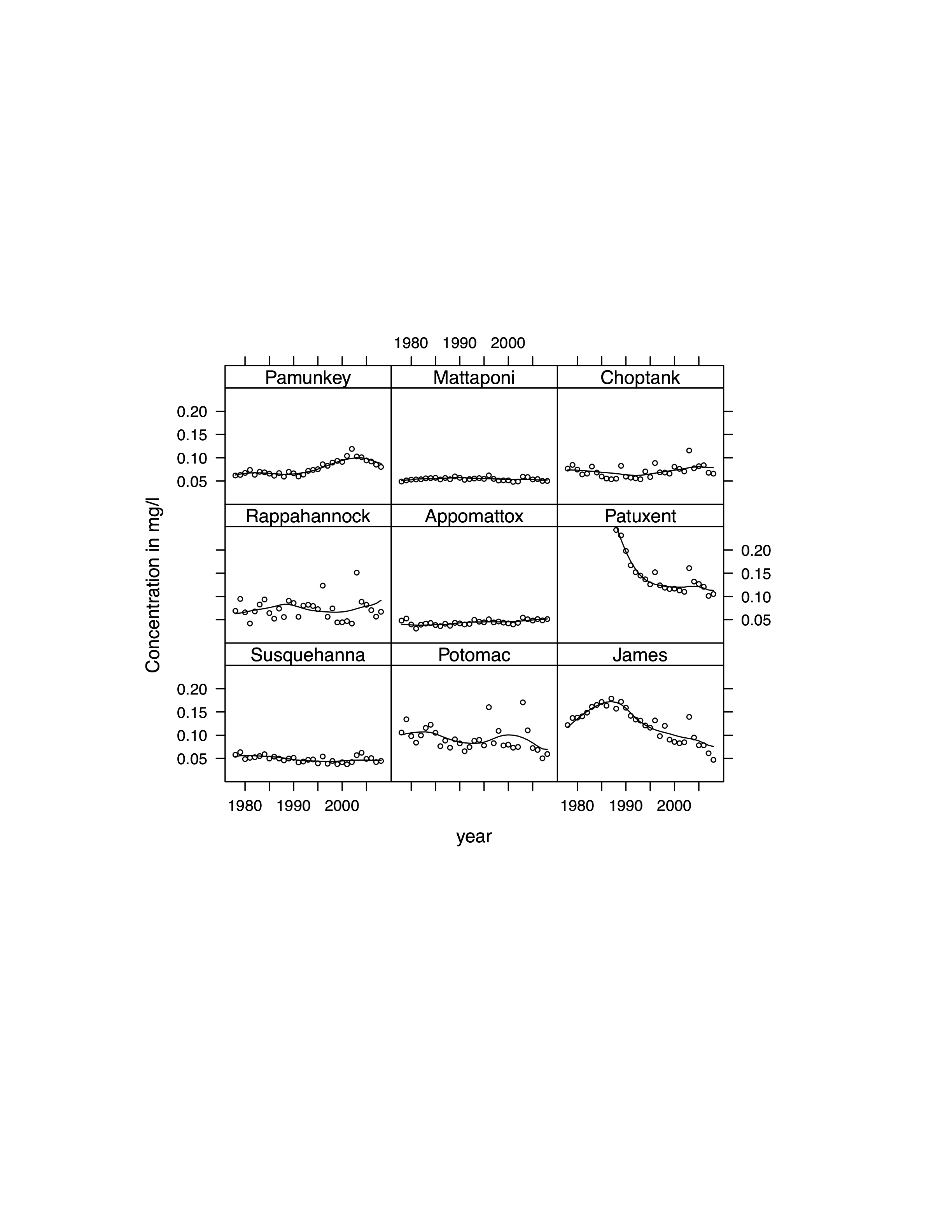


Figure B2: Total Phosphorus concentrations for the nine River Input Monitoring sites using WRTDS method. Circles are the average annual estimates. Line traces through the flow-normalized annual estimates. Patuxent River record is truncated, values above 0.25 mg/l are not shown, but they are shown in the left panel of Figure 9.

| Time period | 1978-2008 | | 2000-2008 | |
| --- | --- | --- | --- | --- |
| River | slope, % per year | concentration change in mg/l | slope, % per year | concentration change in mg/l |
| Susquehanna | -0.5% | -0.01 | +0.3% | +0.00 |
| Potomac | -1.0% | -0.03 | -3.9% | -0.03 |
| James | -1.2% | -0.04 | -3.0% | -0.24 |
| Rappahannock | +1.5% | +0.03 | +4.7% | +0.03 |
| Appomattox | +0.9% | +0.01 | +1.6% | +0.01 |
| Patuxent | -2.9% | -0.74 | -0.7% | -0.01 |
| Pamunkey | +1.3% | +0.02 | -0.9% | -0.01 |
| Mattaponi | +0.1% | +0.00 | -0.2% | -0.00 |
| Choptank | +0.2% | +0.00 | +0.9% | +0.01 |

Table B1: Changes in total phosphorus concentration for the nine RIM sites for two periods 1978-2008 and 2000-2008. Concentration change is the flow-normalized annual concentration estimate at the end of the period minus the flow-normalized annual concentration estimate at the beginning of the period. The slope is this concentration change per year expressed in percentage terms over the period.


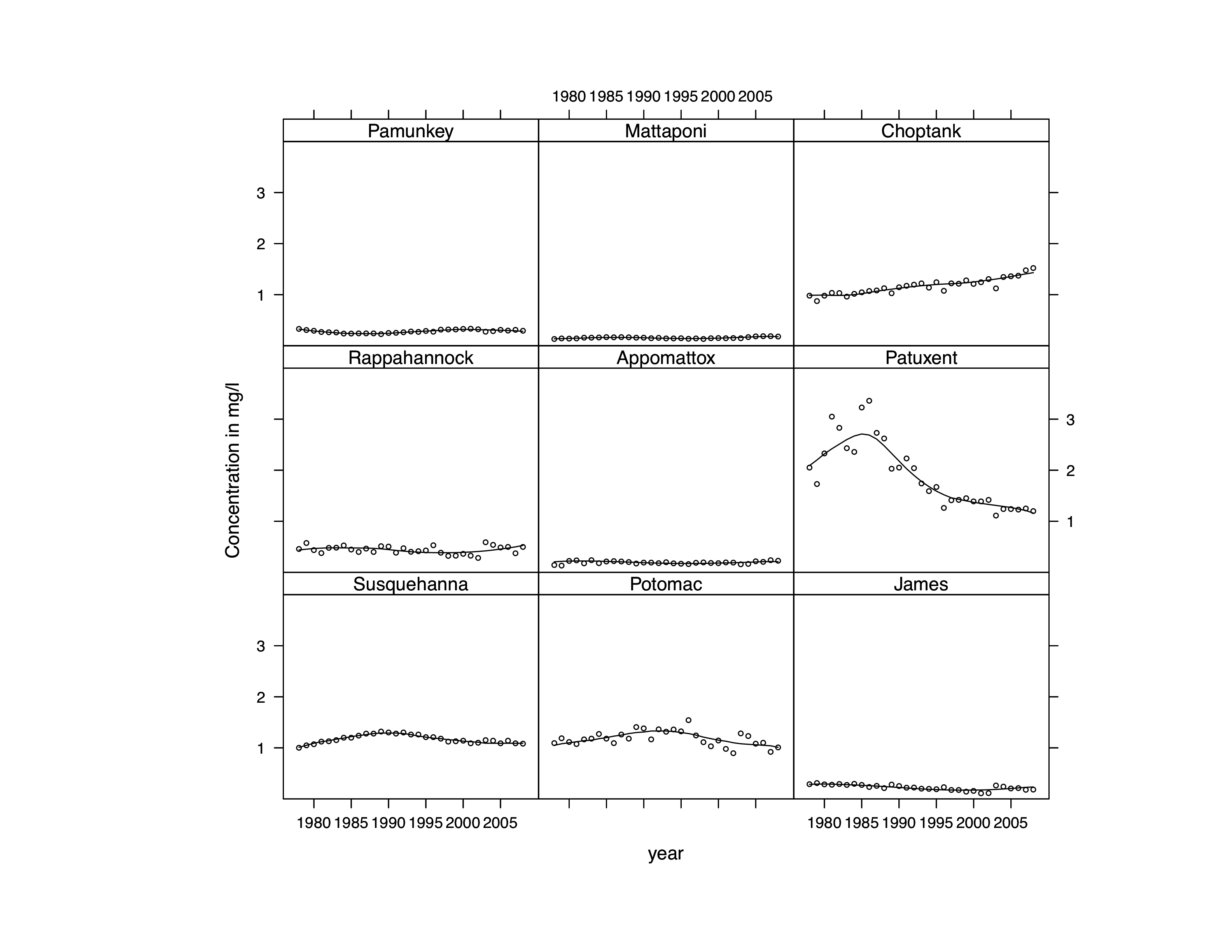


Figure B3: Dissolved nitrate plus nitrite concentrations for the nine River Input Monitoring sites using WRTDS method. Circles are the average annual estimates. Line traces through the flow-normalized annual estimates.

| Time period | 1978-2008 | | 2000-2008 | |
| --- | --- | --- | --- | --- |
| River | slope in % per year | concentration change in mg/l | slope in % per year | concentration change in mg/l |
| Susquehanna | +0.3% | +0.08 | -0.4% | -0.04 |
| Potomac | -0.1% | -0.04 | -1.5% | -0.14 |
| James | -0.6% | -0.05 | +4.4% | +0.06 |
| Rappahannock | +0.7% | +0.09 | +4.3% | +0.14 |
| Appomattox | +0.0% | +0.00 | 1.9% | +0.03 |
| Patuxent | -1.5% | -0.93 | -1.9% | -0.21 |
| Pamunkey | -0.4% | -0.04 | -1.5% | -0.04 |
| Mattaponi | +1.0% | +0.04 | +2.3% | +0.03 |
| Choptank | +1.5% | +0.44 | +1.8% | +0.18 |

Table B2: Changes in dissolved nitrate plus nitrite concentration for the nine RIM sites for two periods 1978-2008 and 2000-2008. Concentration change is the flow normalized annual concentration estimate at the end of the period minus the flow normalized annual concentration estimate at the beginning of the period. The slope is this concentration change per year expressed in percentage terms over the period.
