# Supplementary material for: Weighted Regressions on Time, Discharge, and Season (WRTDS), with an Application to Chesapeake Bay River Inputs
Source: J Am Water Resour Assoc. 2010 Oct;46(5):857–80. doi: 10.1111/j.1752-1688.2010.00482.x (PMC3307614; doi:10.1111/j.1752-1688.2010.00482.x)
Supplement: Supplementary file 3 [file jawr0046-0857-SD3.doc]

| The tables in appendix C provide estimates of concentration in mg/l, and flux (in both lbs/day and kg/day) for all sites, for total phosphorus and for dissolved nitrate plus nitrite, showing annual estimates and flow-normalized annual estimates. The results for flux are shown with more significant figures than are warranted by the method, but because users of these results may wish to convert these to other units, more significant figures are preserved in the tables so that the rounding process does not influence further calculations |
| --- |

| YEAR | SUSQUEHANNA | POTOMAC | JAMES | RAPPAHANNOCK | APPOMATTOX | PATUXENT | PAMUNKEY | MATTAPONI | CHOPTANK |
| --- | --- | --- | --- | --- | --- | --- | --- | --- | --- |
| 1978 | 0.058 | 0.106 | 0.122 | 0.069 | 0.048 | 0.822 | 0.062 | 0.049 | 0.077 |
| 1979 | 0.064 | 0.134 | 0.137 | 0.095 | 0.053 | 0.569 | 0.063 | 0.052 | 0.085 |
| 1980 | 0.049 | 0.098 | 0.138 | 0.066 | 0.040 | 0.707 | 0.067 | 0.053 | 0.075 |
| 1981 | 0.052 | 0.084 | 0.141 | 0.042 | 0.031 | 0.762 | 0.074 | 0.054 | 0.064 |
| 1982 | 0.053 | 0.100 | 0.148 | 0.068 | 0.039 | 0.593 | 0.063 | 0.054 | 0.066 |
| 1983 | 0.055 | 0.116 | 0.161 | 0.083 | 0.042 | 0.476 | 0.070 | 0.056 | 0.081 |
| 1984 | 0.059 | 0.122 | 0.165 | 0.094 | 0.043 | 0.418 | 0.069 | 0.056 | 0.068 |
| 1985 | 0.050 | 0.105 | 0.171 | 0.064 | 0.039 | 0.381 | 0.066 | 0.057 | 0.060 |
| 1986 | 0.054 | 0.076 | 0.164 | 0.052 | 0.036 | 0.321 | 0.061 | 0.054 | 0.056 |
| 1987 | 0.050 | 0.088 | 0.179 | 0.074 | 0.042 | 0.283 | 0.067 | 0.057 | 0.054 |
| 1988 | 0.046 | 0.073 | 0.157 | 0.056 | 0.037 | 0.243 | 0.059 | 0.054 | 0.055 |
| 1989 | 0.050 | 0.091 | 0.172 | 0.091 | 0.043 | 0.231 | 0.070 | 0.060 | 0.083 |
| 1990 | 0.051 | 0.082 | 0.159 | 0.086 | 0.042 | 0.198 | 0.067 | 0.057 | 0.060 |
| 1991 | 0.042 | 0.066 | 0.142 | 0.056 | 0.040 | 0.167 | 0.060 | 0.053 | 0.057 |
| 1992 | 0.044 | 0.074 | 0.134 | 0.080 | 0.041 | 0.152 | 0.064 | 0.054 | 0.056 |
| 1993 | 0.047 | 0.088 | 0.132 | 0.082 | 0.050 | 0.145 | 0.072 | 0.056 | 0.054 |
| 1994 | 0.048 | 0.090 | 0.121 | 0.080 | 0.046 | 0.137 | 0.074 | 0.056 | 0.071 |
| 1995 | 0.040 | 0.078 | 0.116 | 0.073 | 0.044 | 0.126 | 0.076 | 0.055 | 0.059 |
| 1996 | 0.054 | 0.160 | 0.132 | 0.123 | 0.051 | 0.152 | 0.086 | 0.062 | 0.089 |
| 1997 | 0.039 | 0.083 | 0.098 | 0.056 | 0.044 | 0.124 | 0.083 | 0.055 | 0.069 |
| 1998 | 0.045 | 0.109 | 0.120 | 0.074 | 0.046 | 0.119 | 0.090 | 0.051 | 0.068 |
| 1999 | 0.038 | 0.078 | 0.090 | 0.044 | 0.044 | 0.116 | 0.093 | 0.051 | 0.066 |
| 2000 | 0.042 | 0.080 | 0.086 | 0.045 | 0.042 | 0.117 | 0.091 | 0.052 | 0.081 |
| 2001 | 0.037 | 0.073 | 0.083 | 0.047 | 0.040 | 0.113 | 0.104 | 0.048 | 0.076 |
| 2002 | 0.042 | 0.075 | 0.085 | 0.042 | 0.043 | 0.110 | 0.119 | 0.049 | 0.071 |
| 2003 | 0.057 | 0.171 | 0.139 | 0.151 | 0.055 | 0.161 | 0.103 | 0.059 | 0.116 |
| 2004 | 0.062 | 0.110 | 0.095 | 0.089 | 0.051 | 0.132 | 0.101 | 0.059 | 0.077 |
| 2005 | 0.049 | 0.072 | 0.078 | 0.082 | 0.048 | 0.127 | 0.095 | 0.053 | 0.082 |
| 2006 | 0.051 | 0.068 | 0.078 | 0.071 | 0.052 | 0.121 | 0.092 | 0.054 | 0.084 |
| 2007 | 0.042 | 0.050 | 0.061 | 0.057 | 0.049 | 0.101 | 0.085 | 0.050 | 0.068 |
| 2008 | 0.045 | 0.059 | 0.047 | 0.067 | 0.051 | 0.105 | 0.080 | 0.050 | 0.066 |

Table C1: Average Annual Total Phosphorus Concentration Estimates using WRTDS, in mg/l

| YEAR | SUSQUEHANNA | POTOMAC | JAMES | RAPPAHANNOCK | APPOMATTOX | PATUXENT | PAMUNKEY | MATTAPONI | CHOPTANK |
| --- | --- | --- | --- | --- | --- | --- | --- | --- | --- |
| 1978 | 0.054 | 0.101 | 0.117 | 0.063 | 0.040 | 0.854 | 0.063 | 0.051 | 0.074 |
| 1979 | 0.056 | 0.103 | 0.126 | 0.064 | 0.040 | 0.786 | 0.065 | 0.052 | 0.074 |
| 1980 | 0.056 | 0.105 | 0.135 | 0.066 | 0.039 | 0.705 | 0.066 | 0.053 | 0.073 |
| 1981 | 0.056 | 0.106 | 0.144 | 0.069 | 0.038 | 0.626 | 0.067 | 0.054 | 0.073 |
| 1982 | 0.056 | 0.107 | 0.151 | 0.071 | 0.038 | 0.554 | 0.068 | 0.055 | 0.072 |
| 1983 | 0.055 | 0.107 | 0.158 | 0.072 | 0.039 | 0.488 | 0.068 | 0.055 | 0.071 |
| 1984 | 0.055 | 0.106 | 0.163 | 0.074 | 0.039 | 0.428 | 0.067 | 0.055 | 0.070 |
| 1985 | 0.054 | 0.103 | 0.168 | 0.076 | 0.039 | 0.375 | 0.067 | 0.055 | 0.069 |
| 1986 | 0.053 | 0.099 | 0.171 | 0.078 | 0.040 | 0.328 | 0.066 | 0.056 | 0.068 |
| 1987 | 0.052 | 0.095 | 0.172 | 0.081 | 0.040 | 0.288 | 0.065 | 0.056 | 0.067 |
| 1988 | 0.051 | 0.091 | 0.171 | 0.083 | 0.041 | 0.252 | 0.064 | 0.057 | 0.066 |
| 1989 | 0.049 | 0.088 | 0.166 | 0.083 | 0.042 | 0.222 | 0.064 | 0.057 | 0.065 |
| 1990 | 0.048 | 0.085 | 0.158 | 0.081 | 0.043 | 0.196 | 0.065 | 0.057 | 0.063 |
| 1991 | 0.046 | 0.084 | 0.148 | 0.078 | 0.044 | 0.175 | 0.066 | 0.057 | 0.063 |
| 1992 | 0.045 | 0.083 | 0.137 | 0.074 | 0.045 | 0.158 | 0.068 | 0.056 | 0.062 |
| 1993 | 0.045 | 0.082 | 0.128 | 0.072 | 0.045 | 0.145 | 0.070 | 0.056 | 0.062 |
| 1994 | 0.045 | 0.083 | 0.122 | 0.070 | 0.045 | 0.136 | 0.072 | 0.056 | 0.064 |
| 1995 | 0.044 | 0.085 | 0.116 | 0.069 | 0.045 | 0.130 | 0.076 | 0.055 | 0.065 |
| 1996 | 0.044 | 0.089 | 0.112 | 0.068 | 0.046 | 0.126 | 0.079 | 0.055 | 0.067 |
| 1997 | 0.044 | 0.093 | 0.108 | 0.067 | 0.046 | 0.124 | 0.083 | 0.055 | 0.069 |
| 1998 | 0.044 | 0.097 | 0.106 | 0.066 | 0.046 | 0.122 | 0.087 | 0.054 | 0.070 |
| 1999 | 0.044 | 0.100 | 0.103 | 0.066 | 0.046 | 0.121 | 0.091 | 0.054 | 0.072 |
| 2000 | 0.045 | 0.101 | 0.100 | 0.067 | 0.046 | 0.120 | 0.094 | 0.053 | 0.073 |
| 2001 | 0.045 | 0.100 | 0.097 | 0.068 | 0.046 | 0.120 | 0.097 | 0.053 | 0.075 |
| 2002 | 0.046 | 0.098 | 0.095 | 0.071 | 0.047 | 0.120 | 0.099 | 0.053 | 0.077 |
| 2003 | 0.046 | 0.095 | 0.093 | 0.074 | 0.048 | 0.122 | 0.100 | 0.054 | 0.079 |
| 2004 | 0.047 | 0.090 | 0.091 | 0.077 | 0.049 | 0.122 | 0.100 | 0.054 | 0.080 |
| 2005 | 0.046 | 0.084 | 0.088 | 0.079 | 0.050 | 0.121 | 0.098 | 0.053 | 0.081 |
| 2006 | 0.046 | 0.077 | 0.083 | 0.081 | 0.050 | 0.118 | 0.095 | 0.053 | 0.080 |
| 2007 | 0.046 | 0.071 | 0.078 | 0.085 | 0.051 | 0.114 | 0.091 | 0.052 | 0.079 |
| 2008 | 0.046 | 0.069 | 0.076 | 0.092 | 0.052 | 0.113 | 0.088 | 0.052 | 0.079 |

Figure C2: Flow-normalized Average Annual Total Phosphorus Concentrations, in mg/l.

| YEAR | SUSQUEHANNA | POTOMAC | JAMES | RAPPAHANNOCK | APPOMATTOX | PATUXENT | PAMUNKEY | MATTAPONI | CHOPTANK |
| --- | --- | --- | --- | --- | --- | --- | --- | --- | --- |
| 1978 | 24,443 | 14,855 | 8,397 | 1,676 | 881 | 1,244 | 518 | 197 | 105 |
| 1979 | 28,195 | 25,280 | 14,557 | 3,274 | 1,199 | 1,778 | 810 | 288 | 158 |
| 1980 | 11,268 | 8,254 | 6,002 | 1,097 | 436 | 971 | 323 | 116 | 64 |
| 1981 | 12,765 | 3,709 | 2,366 | 214 | 100 | 586 | 98 | 51 | 30 |
| 1982 | 13,022 | 8,530 | 6,923 | 1,132 | 340 | 643 | 317 | 125 | 46 |
| 1983 | 17,059 | 14,671 | 8,237 | 2,343 | 500 | 991 | 492 | 211 | 124 |
| 1984 | 21,854 | 21,455 | 10,476 | 2,752 | 638 | 845 | 692 | 293 | 85 |
| 1985 | 9,587 | 22,666 | 15,657 | 1,217 | 419 | 422 | 499 | 168 | 28 |
| 1986 | 14,775 | 5,869 | 3,871 | 394 | 181 | 344 | 220 | 96 | 41 |
| 1987 | 9,900 | 9,267 | 13,171 | 1,574 | 466 | 451 | 496 | 194 | 43 |
| 1988 | 7,643 | 7,653 | 3,084 | 658 | 169 | 385 | 239 | 111 | 27 |
| 1989 | 13,144 | 8,954 | 11,088 | 2,104 | 421 | 621 | 517 | 209 | 114 |
| 1990 | 15,416 | 6,558 | 8,496 | 1,583 | 338 | 407 | 485 | 198 | 46 |
| 1991 | 7,921 | 5,584 | 6,654 | 744 | 241 | 238 | 220 | 75 | 38 |
| 1992 | 9,157 | 5,391 | 7,178 | 1,771 | 229 | 238 | 250 | 103 | 28 |
| 1993 | 22,130 | 16,021 | 9,362 | 2,669 | 755 | 366 | 655 | 228 | 50 |
| 1994 | 18,684 | 13,779 | 7,717 | 2,229 | 536 | 351 | 673 | 232 | 110 |
| 1995 | 7,136 | 5,376 | 7,193 | 2,607 | 306 | 213 | 392 | 113 | 35 |
| 1996 | 28,786 | 42,536 | 13,312 | 4,419 | 702 | 625 | 893 | 295 | 170 |
| 1997 | 7,067 | 6,536 | 3,765 | 884 | 290 | 279 | 429 | 156 | 66 |
| 1998 | 14,050 | 20,806 | 11,561 | 3,250 | 698 | 304 | 749 | 231 | 87 |
| 1999 | 6,863 | 3,146 | 2,938 | 679 | 231 | 211 | 240 | 72 | 76 |
| 2000 | 9,378 | 4,132 | 2,555 | 356 | 232 | 228 | 268 | 96 | 90 |
| 2001 | 5,646 | 3,934 | 2,009 | 671 | 194 | 208 | 234 | 71 | 98 |
| 2002 | 9,403 | 4,201 | 1,874 | 454 | 168 | 154 | 161 | 39 | 58 |
| 2003 | 23,364 | 37,035 | 16,559 | 5,897 | 1,095 | 809 | 1,156 | 319 | 246 |
| 2004 | 35,105 | 14,484 | 6,095 | 2,029 | 556 | 342 | 687 | 222 | 69 |
| 2005 | 21,309 | 8,144 | 4,378 | 2,357 | 285 | 365 | 440 | 137 | 82 |
| 2006 | 18,986 | 5,770 | 5,730 | 1,212 | 408 | 355 | 475 | 178 | 90 |
| 2007 | 11,708 | 5,082 | 3,533 | 981 | 258 | 198 | 293 | 118 | 71 |
| 2008 | 14,451 | 7,305 | 1,524 | 1,977 | 265 | 240 | 269 | 120 | 52 |

Table C3a: Average Annual Total Phosphorus Flux Estimates using WRTDS, in lbs/day

| YEAR | SUSQUEHANNA | POTOMAC | JAMES | RAPPAHANNOCK | APPOMATTOX | PATUXENT | PAMUNKEY | MATTAPONI | CHOPTANK |
| --- | --- | --- | --- | --- | --- | --- | --- | --- | --- |
| 1978 | 11,087 | 6,738 | 3,809 | 760 | 400 | 564 | 235 | 89 | 48 |
| 1979 | 12,789 | 11,467 | 6,603 | 1,485 | 544 | 806 | 367 | 131 | 72 |
| 1980 | 5,111 | 3,744 | 2,722 | 498 | 198 | 440 | 147 | 53 | 29 |
| 1981 | 5,790 | 1,682 | 1,073 | 97 | 45 | 266 | 44 | 23 | 14 |
| 1982 | 5,907 | 3,869 | 3,140 | 513 | 154 | 292 | 144 | 57 | 21 |
| 1983 | 7,738 | 6,655 | 3,736 | 1,063 | 227 | 450 | 223 | 96 | 56 |
| 1984 | 9,913 | 9,732 | 4,752 | 1,248 | 289 | 383 | 314 | 133 | 39 |
| 1985 | 4,349 | 10,281 | 7,102 | 552 | 190 | 191 | 226 | 76 | 13 |
| 1986 | 6,702 | 2,662 | 1,756 | 179 | 82 | 156 | 100 | 44 | 19 |
| 1987 | 4,491 | 4,203 | 5,974 | 714 | 211 | 205 | 225 | 88 | 20 |
| 1988 | 3,467 | 3,471 | 1,399 | 298 | 77 | 175 | 108 | 50 | 12 |
| 1989 | 5,962 | 4,061 | 5,029 | 954 | 191 | 282 | 235 | 95 | 52 |
| 1990 | 6,993 | 2,975 | 3,854 | 718 | 153 | 185 | 220 | 90 | 21 |
| 1991 | 3,593 | 2,533 | 3,018 | 337 | 109 | 108 | 100 | 34 | 17 |
| 1992 | 4,154 | 2,445 | 3,256 | 803 | 104 | 108 | 113 | 47 | 13 |
| 1993 | 10,038 | 7,267 | 4,247 | 1,211 | 342 | 166 | 297 | 103 | 23 |
| 1994 | 8,475 | 6,250 | 3,500 | 1,011 | 243 | 159 | 305 | 105 | 50 |
| 1995 | 3,237 | 2,439 | 3,263 | 1,183 | 139 | 97 | 178 | 51 | 16 |
| 1996 | 13,057 | 19,294 | 6,038 | 2,004 | 318 | 283 | 405 | 134 | 77 |
| 1997 | 3,206 | 2,965 | 1,708 | 401 | 132 | 127 | 195 | 71 | 30 |
| 1998 | 6,373 | 9,437 | 5,244 | 1,474 | 317 | 138 | 340 | 105 | 39 |
| 1999 | 3,113 | 1,427 | 1,333 | 308 | 105 | 96 | 109 | 33 | 34 |
| 2000 | 4,254 | 1,874 | 1,159 | 161 | 105 | 103 | 122 | 44 | 41 |
| 2001 | 2,561 | 1,784 | 911 | 304 | 88 | 94 | 106 | 32 | 44 |
| 2002 | 4,265 | 1,906 | 850 | 206 | 76 | 70 | 73 | 18 | 26 |
| 2003 | 10,598 | 16,799 | 7,511 | 2,675 | 497 | 367 | 524 | 145 | 112 |
| 2004 | 15,923 | 6,570 | 2,765 | 920 | 252 | 155 | 312 | 101 | 31 |
| 2005 | 9,666 | 3,694 | 1,986 | 1,069 | 129 | 166 | 200 | 62 | 37 |
| 2006 | 8,612 | 2,617 | 2,599 | 550 | 185 | 161 | 215 | 81 | 41 |
| 2007 | 5,311 | 2,305 | 1,603 | 445 | 117 | 90 | 133 | 54 | 32 |
| 2008 | 6,555 | 3,313 | 691 | 897 | 120 | 109 | 122 | 54 | 24 |

Table C3b: Average Annual Total Phosphorus Flux Estimates using WRTDS, in kg/day

| YEAR | SUSQUEHANNA | POTOMAC | JAMES | RAPPAHANNOCK | APPOMATTOX | PATUXENT | PAMUNKEY | MATTAPONI | CHOPTANK |
| --- | --- | --- | --- | --- | --- | --- | --- | --- | --- |
| 1978 | 18,448 | 11,927 | 6,638 | 1,435 | 472 | 1,177 | 392 | 135 | 84 |
| 1979 | 18,211 | 12,358 | 6,999 | 1,380 | 447 | 1,124 | 404 | 142 | 83 |
| 1980 | 17,699 | 12,736 | 7,194 | 1,367 | 431 | 1,052 | 414 | 147 | 82 |
| 1981 | 17,205 | 13,060 | 7,381 | 1,408 | 416 | 979 | 418 | 155 | 81 |
| 1982 | 16,591 | 13,468 | 7,680 | 1,448 | 411 | 909 | 418 | 160 | 80 |
| 1983 | 16,041 | 13,758 | 7,816 | 1,452 | 413 | 841 | 417 | 163 | 78 |
| 1984 | 15,512 | 13,774 | 8,075 | 1,487 | 408 | 774 | 417 | 165 | 77 |
| 1985 | 15,167 | 13,518 | 8,366 | 1,543 | 406 | 713 | 422 | 168 | 76 |
| 1986 | 14,880 | 13,411 | 8,573 | 1,629 | 401 | 653 | 426 | 172 | 76 |
| 1987 | 14,522 | 12,655 | 8,789 | 1,718 | 402 | 599 | 432 | 176 | 75 |
| 1988 | 14,116 | 12,265 | 8,827 | 1,827 | 415 | 539 | 438 | 180 | 73 |
| 1989 | 13,726 | 11,791 | 8,759 | 1,891 | 439 | 483 | 446 | 184 | 72 |
| 1990 | 13,401 | 11,474 | 8,454 | 1,909 | 462 | 430 | 449 | 185 | 71 |
| 1991 | 13,116 | 10,832 | 8,116 | 1,880 | 476 | 389 | 449 | 183 | 69 |
| 1992 | 12,940 | 10,373 | 7,680 | 1,837 | 481 | 354 | 446 | 180 | 69 |
| 1993 | 12,916 | 9,982 | 7,337 | 1,797 | 482 | 328 | 446 | 176 | 69 |
| 1994 | 13,072 | 9,997 | 7,064 | 1,750 | 476 | 312 | 447 | 173 | 70 |
| 1995 | 13,044 | 10,036 | 6,880 | 1,762 | 470 | 302 | 454 | 172 | 71 |
| 1996 | 13,128 | 10,540 | 6,722 | 1,763 | 459 | 296 | 461 | 170 | 72 |
| 1997 | 13,277 | 11,241 | 6,623 | 1,779 | 450 | 294 | 471 | 168 | 74 |
| 1998 | 13,639 | 12,129 | 6,518 | 1,770 | 438 | 293 | 477 | 165 | 75 |
| 1999 | 13,805 | 12,507 | 6,454 | 1,836 | 431 | 292 | 485 | 163 | 77 |
| 2000 | 14,110 | 12,823 | 6,401 | 1,881 | 423 | 292 | 492 | 161 | 79 |
| 2001 | 14,490 | 13,035 | 6,435 | 1,947 | 421 | 294 | 503 | 160 | 81 |
| 2002 | 15,095 | 13,295 | 6,543 | 1,988 | 418 | 300 | 514 | 160 | 84 |
| 2003 | 15,359 | 13,008 | 6,705 | 2,149 | 423 | 307 | 529 | 161 | 86 |
| 2004 | 15,610 | 12,699 | 6,905 | 2,274 | 429 | 313 | 538 | 162 | 88 |
| 2005 | 15,774 | 12,245 | 7,115 | 2,414 | 436 | 313 | 541 | 162 | 89 |
| 2006 | 16,020 | 11,713 | 7,288 | 2,520 | 439 | 306 | 533 | 160 | 89 |
| 2007 | 15,972 | 10,835 | 7,362 | 2,820 | 444 | 299 | 529 | 160 | 90 |
| 2008 | 16,259 | 10,751 | 7,704 | 3,150 | 450 | 296 | 534 | 162 | 91 |

Figure C4a: Flow-normalized Average Annual Total Phosphorus Flux in lbs/day.

| YEAR | SUSQUEHANNA | POTOMAC | JAMES | RAPPAHANNOCK | APPOMATTOX | PATUXENT | PAMUNKEY | MATTAPONI | CHOPTANK |
| --- | --- | --- | --- | --- | --- | --- | --- | --- | --- |
| 1978 | 8,368 | 5,410 | 3,011 | 651 | 214 | 534 | 178 | 61 | 38 |
| 1979 | 8,260 | 5,605 | 3,175 | 626 | 203 | 510 | 183 | 64 | 38 |
| 1980 | 8,028 | 5,777 | 3,263 | 620 | 195 | 477 | 188 | 67 | 37 |
| 1981 | 7,804 | 5,924 | 3,348 | 639 | 189 | 444 | 190 | 70 | 37 |
| 1982 | 7,526 | 6,109 | 3,484 | 657 | 186 | 412 | 190 | 73 | 36 |
| 1983 | 7,276 | 6,241 | 3,545 | 659 | 187 | 381 | 189 | 74 | 35 |
| 1984 | 7,036 | 6,248 | 3,663 | 674 | 185 | 351 | 189 | 75 | 35 |
| 1985 | 6,880 | 6,132 | 3,795 | 700 | 184 | 323 | 191 | 76 | 34 |
| 1986 | 6,749 | 6,083 | 3,889 | 739 | 182 | 296 | 193 | 78 | 34 |
| 1987 | 6,587 | 5,740 | 3,987 | 779 | 182 | 272 | 196 | 80 | 34 |
| 1988 | 6,403 | 5,563 | 4,004 | 829 | 188 | 244 | 199 | 82 | 33 |
| 1989 | 6,226 | 5,348 | 3,973 | 858 | 199 | 219 | 202 | 83 | 33 |
| 1990 | 6,079 | 5,205 | 3,835 | 866 | 210 | 195 | 204 | 84 | 32 |
| 1991 | 5,949 | 4,913 | 3,681 | 853 | 216 | 176 | 204 | 83 | 31 |
| 1992 | 5,869 | 4,705 | 3,484 | 833 | 218 | 161 | 202 | 82 | 31 |
| 1993 | 5,859 | 4,528 | 3,328 | 815 | 219 | 149 | 202 | 80 | 31 |
| 1994 | 5,929 | 4,535 | 3,204 | 794 | 216 | 142 | 203 | 78 | 32 |
| 1995 | 5,917 | 4,552 | 3,121 | 799 | 213 | 137 | 206 | 78 | 32 |
| 1996 | 5,955 | 4,781 | 3,049 | 800 | 208 | 134 | 209 | 77 | 33 |
| 1997 | 6,022 | 5,099 | 3,004 | 807 | 204 | 133 | 214 | 76 | 34 |
| 1998 | 6,187 | 5,502 | 2,957 | 803 | 199 | 133 | 216 | 75 | 34 |
| 1999 | 6,262 | 5,673 | 2,927 | 833 | 195 | 132 | 220 | 74 | 35 |
| 2000 | 6,400 | 5,816 | 2,903 | 853 | 192 | 132 | 223 | 73 | 36 |
| 2001 | 6,573 | 5,913 | 2,919 | 883 | 191 | 133 | 228 | 73 | 37 |
| 2002 | 6,847 | 6,031 | 2,968 | 902 | 190 | 136 | 233 | 73 | 38 |
| 2003 | 6,967 | 5,900 | 3,041 | 975 | 192 | 139 | 240 | 73 | 39 |
| 2004 | 7,081 | 5,760 | 3,132 | 1,031 | 195 | 142 | 244 | 73 | 40 |
| 2005 | 7,155 | 5,554 | 3,227 | 1,095 | 198 | 142 | 245 | 73 | 40 |
| 2006 | 7,267 | 5,313 | 3,306 | 1,143 | 199 | 139 | 242 | 73 | 40 |
| 2007 | 7,245 | 4,915 | 3,339 | 1,279 | 201 | 136 | 240 | 73 | 41 |
| 2008 | 7,375 | 4,877 | 3,494 | 1,429 | 204 | 134 | 242 | 73 | 41 |

Figure C4b: Flow-normalized Average Annual Total Phosphorus Flux in kg/day.

| YEAR | SUSQUEHANNA | POTOMAC | JAMES | RAPPAHANNOCK | APPOMATTOX | PATUXENT | PAMUNKEY | MATTAPONI | CHOPTANK |
| --- | --- | --- | --- | --- | --- | --- | --- | --- | --- |
| 1978 | 1.00 | 1.09 | 0.29 | 0.46 | 0.14 | 2.05 | 0.33 | 0.13 | 0.98 |
| 1979 | 1.05 | 1.19 | 0.31 | 0.57 | 0.13 | 1.73 | 0.31 | 0.14 | 0.87 |
| 1980 | 1.07 | 1.12 | 0.29 | 0.44 | 0.22 | 2.33 | 0.29 | 0.14 | 0.98 |
| 1981 | 1.12 | 1.07 | 0.28 | 0.38 | 0.23 | 3.05 | 0.27 | 0.14 | 1.04 |
| 1982 | 1.13 | 1.17 | 0.29 | 0.48 | 0.18 | 2.83 | 0.27 | 0.16 | 1.03 |
| 1983 | 1.15 | 1.18 | 0.27 | 0.48 | 0.24 | 2.43 | 0.26 | 0.16 | 0.96 |
| 1984 | 1.20 | 1.27 | 0.30 | 0.53 | 0.18 | 2.36 | 0.24 | 0.16 | 1.02 |
| 1985 | 1.20 | 1.18 | 0.27 | 0.45 | 0.21 | 3.23 | 0.24 | 0.17 | 1.05 |
| 1986 | 1.24 | 1.09 | 0.23 | 0.40 | 0.22 | 3.36 | 0.24 | 0.16 | 1.07 |
| 1987 | 1.28 | 1.26 | 0.26 | 0.46 | 0.21 | 2.73 | 0.24 | 0.17 | 1.08 |
| 1988 | 1.28 | 1.18 | 0.21 | 0.40 | 0.20 | 2.62 | 0.24 | 0.16 | 1.13 |
| 1989 | 1.32 | 1.41 | 0.28 | 0.51 | 0.17 | 2.03 | 0.23 | 0.16 | 1.03 |
| 1990 | 1.30 | 1.38 | 0.25 | 0.51 | 0.19 | 2.05 | 0.25 | 0.16 | 1.15 |
| 1991 | 1.28 | 1.17 | 0.22 | 0.38 | 0.19 | 2.23 | 0.25 | 0.15 | 1.18 |
| 1992 | 1.30 | 1.36 | 0.22 | 0.47 | 0.18 | 2.04 | 0.26 | 0.15 | 1.20 |
| 1993 | 1.26 | 1.32 | 0.20 | 0.40 | 0.20 | 1.74 | 0.28 | 0.14 | 1.22 |
| 1994 | 1.26 | 1.36 | 0.20 | 0.41 | 0.17 | 1.59 | 0.27 | 0.14 | 1.14 |
| 1995 | 1.21 | 1.32 | 0.19 | 0.43 | 0.17 | 1.67 | 0.29 | 0.15 | 1.25 |
| 1996 | 1.21 | 1.54 | 0.23 | 0.53 | 0.16 | 1.26 | 0.27 | 0.14 | 1.08 |
| 1997 | 1.18 | 1.24 | 0.18 | 0.38 | 0.19 | 1.41 | 0.31 | 0.14 | 1.22 |
| 1998 | 1.12 | 1.11 | 0.18 | 0.32 | 0.19 | 1.42 | 0.32 | 0.13 | 1.21 |
| 1999 | 1.13 | 1.03 | 0.14 | 0.33 | 0.18 | 1.45 | 0.32 | 0.15 | 1.28 |
| 2000 | 1.14 | 1.14 | 0.16 | 0.36 | 0.18 | 1.39 | 0.33 | 0.15 | 1.21 |
| 2001 | 1.09 | 0.98 | 0.11 | 0.32 | 0.19 | 1.39 | 0.33 | 0.15 | 1.25 |
| 2002 | 1.10 | 0.89 | 0.11 | 0.28 | 0.19 | 1.42 | 0.32 | 0.15 | 1.31 |
| 2003 | 1.15 | 1.28 | 0.26 | 0.59 | 0.16 | 1.11 | 0.27 | 0.15 | 1.12 |
| 2004 | 1.14 | 1.23 | 0.24 | 0.54 | 0.16 | 1.24 | 0.29 | 0.17 | 1.35 |
| 2005 | 1.09 | 1.08 | 0.20 | 0.49 | 0.22 | 1.24 | 0.31 | 0.18 | 1.36 |
| 2006 | 1.14 | 1.10 | 0.21 | 0.50 | 0.21 | 1.23 | 0.30 | 0.19 | 1.37 |
| 2007 | 1.09 | 0.92 | 0.18 | 0.37 | 0.24 | 1.25 | 0.31 | 0.19 | 1.48 |
| 2008 | 1.08 | 1.01 | 0.18 | 0.50 | 0.23 | 1.20 | 0.29 | 0.18 | 1.52 |

Table C5: Average Annual Dissolved Nitrate plus Nitrite Concentration Estimates using WRTDS, in mg/l

| YEAR | SUSQUEHANNA | POTOMAC | JAMES | RAPPAHANNOCK | APPOMATTOX | PATUXENT | PAMUNKEY | MATTAPONI | CHOPTANK |
| --- | --- | --- | --- | --- | --- | --- | --- | --- | --- |
| 1978 | 1.01 | 1.05 | 0.29 | 0.44 | 0.21 | 2.09 | 0.33 | 0.14 | 0.99 |
| 1979 | 1.05 | 1.08 | 0.29 | 0.45 | 0.22 | 2.20 | 0.31 | 0.14 | 0.99 |
| 1980 | 1.09 | 1.10 | 0.29 | 0.47 | 0.22 | 2.32 | 0.29 | 0.14 | 0.99 |
| 1981 | 1.12 | 1.12 | 0.29 | 0.47 | 0.22 | 2.42 | 0.28 | 0.15 | 0.99 |
| 1982 | 1.14 | 1.14 | 0.28 | 0.48 | 0.22 | 2.51 | 0.26 | 0.15 | 0.99 |
| 1983 | 1.17 | 1.15 | 0.28 | 0.48 | 0.22 | 2.60 | 0.25 | 0.16 | 0.99 |
| 1984 | 1.19 | 1.18 | 0.27 | 0.48 | 0.22 | 2.67 | 0.24 | 0.16 | 1.00 |
| 1985 | 1.22 | 1.20 | 0.27 | 0.48 | 0.21 | 2.71 | 0.24 | 0.16 | 1.02 |
| 1986 | 1.24 | 1.23 | 0.26 | 0.48 | 0.21 | 2.69 | 0.24 | 0.16 | 1.04 |
| 1987 | 1.26 | 1.25 | 0.25 | 0.48 | 0.21 | 2.61 | 0.24 | 0.16 | 1.07 |
| 1988 | 1.28 | 1.28 | 0.24 | 0.47 | 0.20 | 2.48 | 0.24 | 0.16 | 1.09 |
| 1989 | 1.29 | 1.30 | 0.23 | 0.46 | 0.19 | 2.33 | 0.24 | 0.16 | 1.11 |
| 1990 | 1.29 | 1.31 | 0.22 | 0.45 | 0.19 | 2.18 | 0.24 | 0.16 | 1.13 |
| 1991 | 1.29 | 1.33 | 0.21 | 0.43 | 0.18 | 2.03 | 0.25 | 0.15 | 1.14 |
| 1992 | 1.28 | 1.33 | 0.20 | 0.42 | 0.18 | 1.90 | 0.26 | 0.15 | 1.16 |
| 1993 | 1.26 | 1.33 | 0.20 | 0.40 | 0.18 | 1.78 | 0.27 | 0.15 | 1.18 |
| 1994 | 1.23 | 1.32 | 0.19 | 0.39 | 0.17 | 1.68 | 0.28 | 0.14 | 1.19 |
| 1995 | 1.21 | 1.30 | 0.18 | 0.39 | 0.17 | 1.59 | 0.29 | 0.14 | 1.20 |
| 1996 | 1.19 | 1.28 | 0.18 | 0.39 | 0.17 | 1.52 | 0.29 | 0.14 | 1.21 |
| 1997 | 1.18 | 1.25 | 0.17 | 0.38 | 0.17 | 1.46 | 0.30 | 0.14 | 1.21 |
| 1998 | 1.16 | 1.21 | 0.17 | 0.38 | 0.18 | 1.43 | 0.31 | 0.14 | 1.22 |
| 1999 | 1.15 | 1.18 | 0.17 | 0.39 | 0.18 | 1.40 | 0.32 | 0.15 | 1.23 |
| 2000 | 1.13 | 1.15 | 0.17 | 0.39 | 0.18 | 1.37 | 0.32 | 0.15 | 1.25 |
| 2001 | 1.12 | 1.13 | 0.18 | 0.40 | 0.18 | 1.35 | 0.32 | 0.15 | 1.27 |
| 2002 | 1.11 | 1.10 | 0.18 | 0.41 | 0.19 | 1.33 | 0.32 | 0.16 | 1.29 |
| 2003 | 1.09 | 1.08 | 0.19 | 0.42 | 0.19 | 1.31 | 0.31 | 0.16 | 1.31 |
| 2004 | 1.09 | 1.07 | 0.19 | 0.44 | 0.20 | 1.29 | 0.31 | 0.17 | 1.33 |
| 2005 | 1.09 | 1.06 | 0.20 | 0.46 | 0.20 | 1.27 | 0.30 | 0.18 | 1.36 |
| 2006 | 1.09 | 1.05 | 0.22 | 0.48 | 0.21 | 1.24 | 0.30 | 0.18 | 1.38 |
| 2007 | 1.09 | 1.04 | 0.23 | 0.50 | 0.21 | 1.21 | 0.29 | 0.18 | 1.41 |
| 2008 | 1.09 | 1.01 | 0.23 | 0.53 | 0.21 | 1.16 | 0.28 | 0.18 | 1.43 |

Figure C6: Flow-normalized Average Annual Dissolved Nitrate Plus Nitrite Concentrations, in mg/l.

| YEAR | SUSQUEHANNA | POTOMAC | JAMES | RAPPAHANNOCK | APPOMATTOX | PATUXENT | PAMUNKEY | MATTAPONI | CHOPTANK |
| --- | --- | --- | --- | --- | --- | --- | --- | --- | --- |
| 1978 | 256,001 | 92,587 | 14,153 | 5,776 | 1,548 | 3,651 | 2,328 | 550 | 693 |
| 1979 | 307,297 | 138,502 | 20,731 | 10,265 | 1,985 | 5,223 | 3,032 | 735 | 879 |
| 1980 | 172,418 | 75,839 | 11,857 | 5,124 | 1,214 | 3,518 | 1,371 | 371 | 619 |
| 1981 | 191,632 | 41,186 | 5,230 | 1,705 | 491 | 2,361 | 399 | 154 | 387 |
| 1982 | 221,872 | 77,653 | 12,271 | 4,777 | 1,162 | 3,012 | 1,222 | 375 | 603 |
| 1983 | 270,239 | 107,609 | 13,833 | 7,370 | 1,536 | 4,477 | 1,611 | 588 | 934 |
| 1984 | 323,140 | 122,008 | 15,661 | 7,895 | 1,767 | 4,341 | 2,041 | 778 | 796 |
| 1985 | 212,792 | 83,202 | 12,121 | 4,744 | 1,302 | 3,042 | 1,298 | 470 | 382 |
| 1986 | 289,281 | 62,764 | 6,347 | 2,823 | 794 | 3,007 | 789 | 322 | 541 |
| 1987 | 229,790 | 86,816 | 13,970 | 4,808 | 1,572 | 3,488 | 1,439 | 550 | 604 |
| 1988 | 196,867 | 62,428 | 5,190 | 2,931 | 725 | 3,417 | 827 | 350 | 466 |
| 1989 | 270,723 | 89,979 | 14,869 | 5,182 | 1,323 | 4,185 | 1,407 | 480 | 1,086 |
| 1990 | 348,837 | 84,697 | 13,135 | 5,644 | 1,109 | 3,531 | 1,420 | 505 | 677 |
| 1991 | 226,060 | 76,701 | 10,258 | 3,673 | 795 | 2,700 | 738 | 232 | 613 |
| 1992 | 253,113 | 79,055 | 10,214 | 5,601 | 755 | 2,526 | 837 | 279 | 545 |
| 1993 | 364,863 | 137,249 | 12,886 | 7,143 | 1,759 | 3,319 | 1,816 | 546 | 709 |
| 1994 | 353,800 | 138,806 | 11,799 | 6,212 | 1,345 | 3,271 | 1,942 | 551 | 1,057 |
| 1995 | 196,710 | 75,786 | 9,202 | 4,947 | 863 | 2,204 | 1,154 | 294 | 611 |
| 1996 | 429,131 | 227,608 | 16,073 | 10,160 | 1,640 | 4,178 | 2,191 | 603 | 1,418 |
| 1997 | 207,820 | 81,809 | 7,611 | 4,208 | 933 | 2,767 | 1,375 | 417 | 871 |
| 1998 | 278,227 | 144,590 | 15,248 | 7,449 | 1,752 | 2,747 | 2,107 | 603 | 878 |
| 1999 | 184,813 | 46,680 | 4,925 | 2,564 | 665 | 1,949 | 701 | 191 | 720 |
| 2000 | 217,392 | 53,333 | 5,097 | 2,453 | 755 | 2,243 | 938 | 284 | 960 |
| 2001 | 149,999 | 47,213 | 3,383 | 2,690 | 565 | 2,084 | 729 | 216 | 872 |
| 2002 | 207,760 | 46,083 | 3,889 | 2,171 | 549 | 1,585 | 421 | 127 | 619 |
| 2003 | 374,758 | 176,277 | 21,135 | 12,584 | 2,223 | 3,928 | 2,553 | 696 | 1,710 |
| 2004 | 399,440 | 109,408 | 11,877 | 6,817 | 1,401 | 2,680 | 1,647 | 584 | 829 |
| 2005 | 299,281 | 72,338 | 8,554 | 6,108 | 949 | 2,503 | 1,258 | 471 | 963 |
| 2006 | 295,688 | 67,680 | 9,381 | 4,934 | 1,025 | 2,266 | 1,216 | 484 | 937 |
| 2007 | 226,046 | 56,804 | 6,879 | 3,084 | 800 | 1,801 | 862 | 439 | 769 |
| 2008 | 249,404 | 68,429 | 4,587 | 3,245 | 643 | 1,798 | 625 | 300 | 696 |

Table C7a: Average Annual Dissolved Nitrate plus Nitrite Flux Estimates using WRTDS, in lbs/day

| YEAR | SUSQUEHANNA | POTOMAC | JAMES | RAPPAHANNOCK | APPOMATTOX | PATUXENT | PAMUNKEY | MATTAPONI | CHOPTANK |
| --- | --- | --- | --- | --- | --- | --- | --- | --- | --- |
| 1978 | 116,120 | 41,997 | 6,420 | 2,620 | 702 | 1,656 | 1,056 | 249 | 314 |
| 1979 | 139,388 | 62,823 | 9,403 | 4,656 | 900 | 2,369 | 1,375 | 333 | 399 |
| 1980 | 78,207 | 34,400 | 5,378 | 2,324 | 551 | 1,596 | 622 | 168 | 281 |
| 1981 | 86,923 | 18,682 | 2,372 | 773 | 223 | 1,071 | 181 | 70 | 176 |
| 1982 | 100,639 | 35,223 | 5,566 | 2,167 | 527 | 1,366 | 554 | 170 | 274 |
| 1983 | 122,578 | 48,811 | 6,275 | 3,343 | 697 | 2,031 | 731 | 267 | 424 |
| 1984 | 146,574 | 55,342 | 7,104 | 3,581 | 801 | 1,969 | 926 | 353 | 361 |
| 1985 | 96,521 | 37,740 | 5,498 | 2,152 | 591 | 1,380 | 589 | 213 | 173 |
| 1986 | 131,216 | 28,469 | 2,879 | 1,280 | 360 | 1,364 | 358 | 146 | 245 |
| 1987 | 104,231 | 39,379 | 6,337 | 2,181 | 713 | 1,582 | 653 | 249 | 274 |
| 1988 | 89,297 | 28,317 | 2,354 | 1,329 | 329 | 1,550 | 375 | 159 | 211 |
| 1989 | 122,798 | 40,814 | 6,744 | 2,351 | 600 | 1,898 | 638 | 218 | 493 |
| 1990 | 158,230 | 38,418 | 5,958 | 2,560 | 503 | 1,602 | 644 | 229 | 307 |
| 1991 | 102,539 | 34,791 | 4,653 | 1,666 | 361 | 1,225 | 335 | 105 | 278 |
| 1992 | 114,810 | 35,859 | 4,633 | 2,541 | 342 | 1,146 | 380 | 127 | 247 |
| 1993 | 165,499 | 62,255 | 5,845 | 3,240 | 798 | 1,505 | 824 | 248 | 322 |
| 1994 | 160,481 | 62,961 | 5,352 | 2,818 | 610 | 1,484 | 881 | 250 | 479 |
| 1995 | 89,226 | 34,376 | 4,174 | 2,244 | 391 | 1,000 | 523 | 133 | 277 |
| 1996 | 194,651 | 103,241 | 7,291 | 4,608 | 744 | 1,895 | 994 | 274 | 643 |
| 1997 | 94,266 | 37,108 | 3,452 | 1,909 | 423 | 1,255 | 624 | 189 | 395 |
| 1998 | 126,202 | 65,585 | 6,916 | 3,379 | 795 | 1,246 | 956 | 274 | 398 |
| 1999 | 83,830 | 21,174 | 2,234 | 1,163 | 302 | 884 | 318 | 87 | 327 |
| 2000 | 98,607 | 24,191 | 2,312 | 1,113 | 342 | 1,017 | 425 | 129 | 435 |
| 2001 | 68,038 | 21,415 | 1,535 | 1,220 | 256 | 945 | 331 | 98 | 396 |
| 2002 | 94,238 | 20,903 | 1,764 | 985 | 249 | 719 | 191 | 58 | 281 |
| 2003 | 169,987 | 79,958 | 9,587 | 5,708 | 1,008 | 1,782 | 1,158 | 316 | 776 |
| 2004 | 181,183 | 49,627 | 5,387 | 3,092 | 635 | 1,216 | 747 | 265 | 376 |
| 2005 | 135,752 | 32,812 | 3,880 | 2,771 | 430 | 1,135 | 571 | 214 | 437 |
| 2006 | 134,122 | 30,699 | 4,255 | 2,238 | 465 | 1,028 | 552 | 220 | 425 |
| 2007 | 102,533 | 25,766 | 3,120 | 1,399 | 363 | 817 | 391 | 199 | 349 |
| 2008 | 113,128 | 31,039 | 2,081 | 1,472 | 292 | 816 | 283 | 136 | 316 |

Table C7b: Average Annual Dissolved Nitrate plus Nitrite Flux Estimates using WRTDS, in kg/day

| YEAR | SUSQUEHANNA | POTOMAC | JAMES | RAPPAHANNOCK | APPOMATTOX | PATUXENT | PAMUNKEY | MATTAPONI | CHOPTANK |
| --- | --- | --- | --- | --- | --- | --- | --- | --- | --- |
| 1978 | 229,087 | 82,438 | 12,238 | 5,161 | 981 | 3,312 | 1,765 | 400 | 618 |
| 1979 | 238,441 | 83,828 | 12,296 | 5,295 | 1,040 | 3,446 | 1,673 | 408 | 630 |
| 1980 | 246,121 | 85,066 | 12,249 | 5,449 | 1,099 | 3,573 | 1,572 | 420 | 644 |
| 1981 | 252,602 | 86,402 | 12,122 | 5,554 | 1,141 | 3,676 | 1,500 | 433 | 657 |
| 1982 | 257,929 | 87,650 | 12,048 | 5,600 | 1,198 | 3,771 | 1,435 | 454 | 667 |
| 1983 | 262,859 | 88,666 | 11,915 | 5,565 | 1,238 | 3,853 | 1,345 | 471 | 678 |
| 1984 | 267,964 | 89,770 | 11,800 | 5,551 | 1,269 | 3,928 | 1,277 | 485 | 692 |
| 1985 | 273,494 | 91,091 | 11,689 | 5,559 | 1,300 | 3,982 | 1,255 | 493 | 710 |
| 1986 | 278,666 | 92,297 | 11,541 | 5,554 | 1,317 | 3,991 | 1,244 | 493 | 728 |
| 1987 | 282,802 | 93,478 | 11,396 | 5,570 | 1,326 | 3,931 | 1,237 | 491 | 745 |
| 1988 | 286,234 | 95,105 | 11,215 | 5,560 | 1,317 | 3,795 | 1,227 | 481 | 760 |
| 1989 | 288,628 | 96,816 | 11,048 | 5,518 | 1,295 | 3,627 | 1,229 | 467 | 774 |
| 1990 | 289,495 | 98,083 | 10,835 | 5,425 | 1,261 | 3,448 | 1,236 | 450 | 784 |
| 1991 | 288,591 | 99,030 | 10,611 | 5,316 | 1,228 | 3,286 | 1,254 | 435 | 792 |
| 1992 | 286,780 | 100,070 | 10,330 | 5,198 | 1,196 | 3,130 | 1,270 | 422 | 796 |
| 1993 | 283,973 | 101,301 | 10,064 | 5,097 | 1,171 | 2,987 | 1,289 | 412 | 801 |
| 1994 | 279,666 | 101,637 | 9,799 | 4,996 | 1,140 | 2,852 | 1,300 | 400 | 804 |
| 1995 | 274,997 | 100,393 | 9,615 | 4,984 | 1,126 | 2,737 | 1,316 | 393 | 807 |
| 1996 | 271,713 | 98,570 | 9,480 | 4,994 | 1,117 | 2,642 | 1,329 | 390 | 812 |
| 1997 | 269,694 | 96,818 | 9,431 | 5,028 | 1,124 | 2,572 | 1,350 | 393 | 818 |
| 1998 | 267,396 | 94,844 | 9,390 | 5,035 | 1,128 | 2,518 | 1,365 | 398 | 824 |
| 1999 | 264,708 | 92,506 | 9,400 | 5,102 | 1,143 | 2,481 | 1,382 | 406 | 831 |
| 2000 | 262,152 | 90,367 | 9,426 | 5,156 | 1,149 | 2,444 | 1,386 | 415 | 838 |
| 2001 | 259,785 | 88,614 | 9,517 | 5,228 | 1,158 | 2,407 | 1,387 | 424 | 849 |
| 2002 | 257,199 | 86,979 | 9,623 | 5,288 | 1,156 | 2,365 | 1,378 | 433 | 859 |
| 2003 | 254,320 | 85,310 | 9,808 | 5,420 | 1,166 | 2,329 | 1,374 | 444 | 871 |
| 2004 | 252,610 | 84,242 | 10,030 | 5,533 | 1,171 | 2,290 | 1,366 | 457 | 885 |
| 2005 | 252,122 | 83,957 | 10,310 | 5,611 | 1,178 | 2,255 | 1,363 | 473 | 904 |
| 2006 | 251,433 | 83,787 | 10,491 | 5,551 | 1,154 | 2,214 | 1,344 | 483 | 922 |
| 2007 | 248,668 | 82,477 | 10,532 | 5,447 | 1,109 | 2,165 | 1,303 | 476 | 934 |
| 2008 | 243,205 | 79,711 | 10,299 | 5,313 | 1,028 | 2,081 | 1,226 | 438 | 943 |

Figure C8a: Flow-normalized Average Annual Dissolved Nitrate plus Nitrite Flux in lbs/day.

| YEAR | SUSQUEHANNA | POTOMAC | JAMES | RAPPAHANNOCK | APPOMATTOX | PATUXENT | PAMUNKEY | MATTAPONI | CHOPTANK |
| --- | --- | --- | --- | --- | --- | --- | --- | --- | --- |
| 1978 | 103,912 | 37,393 | 5,551 | 2,341 | 445 | 1,502 | 801 | 181 | 280 |
| 1979 | 108,155 | 38,024 | 5,577 | 2,402 | 472 | 1,563 | 759 | 185 | 286 |
| 1980 | 111,639 | 38,585 | 5,556 | 2,472 | 498 | 1,621 | 713 | 191 | 292 |
| 1981 | 114,578 | 39,191 | 5,498 | 2,519 | 518 | 1,667 | 680 | 196 | 298 |
| 1982 | 116,995 | 39,757 | 5,465 | 2,540 | 543 | 1,710 | 651 | 206 | 303 |
| 1983 | 119,231 | 40,218 | 5,405 | 2,524 | 562 | 1,748 | 610 | 214 | 308 |
| 1984 | 121,546 | 40,719 | 5,352 | 2,518 | 576 | 1,782 | 579 | 220 | 314 |
| 1985 | 124,055 | 41,318 | 5,302 | 2,522 | 590 | 1,806 | 569 | 224 | 322 |
| 1986 | 126,401 | 41,865 | 5,235 | 2,519 | 597 | 1,810 | 564 | 224 | 330 |
| 1987 | 128,277 | 42,401 | 5,169 | 2,527 | 601 | 1,783 | 561 | 223 | 338 |
| 1988 | 129,834 | 43,139 | 5,087 | 2,522 | 597 | 1,721 | 557 | 218 | 345 |
| 1989 | 130,919 | 43,915 | 5,011 | 2,503 | 587 | 1,645 | 557 | 212 | 351 |
| 1990 | 131,313 | 44,490 | 4,915 | 2,461 | 572 | 1,564 | 561 | 204 | 356 |
| 1991 | 130,903 | 44,919 | 4,813 | 2,411 | 557 | 1,491 | 569 | 197 | 359 |
| 1992 | 130,081 | 45,391 | 4,686 | 2,358 | 542 | 1,420 | 576 | 191 | 361 |
| 1993 | 128,808 | 45,949 | 4,565 | 2,312 | 531 | 1,355 | 585 | 187 | 363 |
| 1994 | 126,854 | 46,102 | 4,445 | 2,266 | 517 | 1,294 | 590 | 181 | 365 |
| 1995 | 124,737 | 45,537 | 4,361 | 2,261 | 511 | 1,241 | 597 | 178 | 366 |
| 1996 | 123,247 | 44,711 | 4,300 | 2,265 | 507 | 1,198 | 603 | 177 | 368 |
| 1997 | 122,331 | 43,916 | 4,278 | 2,281 | 510 | 1,167 | 612 | 178 | 371 |
| 1998 | 121,289 | 43,021 | 4,259 | 2,284 | 512 | 1,142 | 619 | 181 | 374 |
| 1999 | 120,070 | 41,960 | 4,264 | 2,314 | 518 | 1,125 | 627 | 184 | 377 |
| 2000 | 118,910 | 40,990 | 4,276 | 2,339 | 521 | 1,109 | 629 | 188 | 380 |
| 2001 | 117,836 | 40,195 | 4,317 | 2,371 | 525 | 1,092 | 629 | 192 | 385 |
| 2002 | 116,664 | 39,453 | 4,365 | 2,399 | 524 | 1,073 | 625 | 196 | 390 |
| 2003 | 115,358 | 38,696 | 4,449 | 2,458 | 529 | 1,056 | 623 | 201 | 395 |
| 2004 | 114,582 | 38,212 | 4,550 | 2,510 | 531 | 1,039 | 620 | 207 | 401 |
| 2005 | 114,361 | 38,082 | 4,677 | 2,545 | 534 | 1,023 | 618 | 215 | 410 |
| 2006 | 114,048 | 38,005 | 4,759 | 2,518 | 523 | 1,004 | 610 | 219 | 418 |
| 2007 | 112,794 | 37,411 | 4,777 | 2,471 | 503 | 982 | 591 | 216 | 424 |
| 2008 | 110,316 | 36,156 | 4,672 | 2,410 | 466 | 944 | 556 | 199 | 428 |

Figure C8b: Flow-normalized Average Annual Dissolved Nitrate plus Nitrite Flux in kg/day.
